# Genomic diversity and evolution analysis of severe fever with thrombocytopenia syndrome in East Asia from 2010 to 2022

**DOI:** 10.3389/fmicb.2023.1233693

**Published:** 2023-08-21

**Authors:** Yao Wang, Bo Pang, Zequn Wang, Xueying Tian, Xiaoying Xu, Xiaowen Chong, Hao Liang, Wei Ma, Zengqiang Kou, Hongling Wen

**Affiliations:** ^1^Department of Epidemiology, School of Public Health, Cheeloo College of Medicine, Shandong University, Jinan, China; ^2^Department of Microbiological Laboratory Technology, School of Public Health, Cheeloo College of Medicine, Shandong University, Jinan, China; ^3^Infection Disease Control of Institute, Shandong Center for Disease Control and Prevention, Shandong Provincial Key Laboratory of Infectious Disease Prevention and Control, Jinan, China

**Keywords:** severe fever with thrombocytopenia syndrome, evolution, reassortment, recombination, selection pressure

## Abstract

**Background:**

Conducting an up-to-date analysis on the genomic diversity and evolution patterns of severe fever with thrombocytopenia syndrome virus (SFTSV) is crucial for elucidating the underlying mechanisms of its emergency and pathogenicity, as well as assessing the extent of its threat to public health.

**Methods:**

Complete genome sequences of SFTSV were obtained from GenBank until December 19, 2022. A thorough phylogenetic analysis was conducted using comprehensive bioinformatics methods to estimate the genomic diversity and evolution.

**Results:**

The phylogenetic classification of SFTSV strains yielded seven lineages (A-G) for each genome segment. SFTSV displayed notable variations in evolutionary patterns among different regions and segments, without a linear accumulation of nucleotide substitutions within segments and regions. The comprehensive analysis revealed 54 recombination events and 17 reassortment strains, including the first discovery of recombination events involving sea-crossing and species-crossing. Selection analysis identified three positive sites (2, 671, 1353) in RNA-dependent RNA polymerase, three positive sites (22, 298, 404) in glycoprotein, and two positive sites (9, 289) in nonstructural protein. No positive selection sites were found in nucleoprotein.

**Conclusion:**

Our study unveiled the existence of multiple evolutionary forces influencing SFTSV, contributing to its increasing genetic diversity, which had the potential to modify its antigenicity and pathogenicity. Furthermore, our study highlights the importance of tracking the spread of SFTSV across regions and species.

## Introduction

Severe fever with thrombocytopenia syndrome (SFTS) is an emerging tick-borne disease caused by the SFTS virus (SFTSV). The International Committee on Taxonomy of Viruses named the virus Dabie bandavirus in 2019, which belongs to *Bandavirus* genus, *Phenuiviridae* family, *Bunyavirales* order (Luo et al., 2023). But SFTSV is currently the most widely used in the world. Initially identified in Henan Province, China in 2009, SFTSV has since been found to be widespread across 23 provinces (Yu et al., 2011; Chen et al., 2022). The occurrence of SFTS or SFTS-like cases outside of China has also been reported in South Korea (Kim et al., 2018), Japan (Takahashi et al., 2014), Australia (Wang et al., 2014), and the United States (McMullan et al., 2012), indicating a potential global distribution of SFTS or similar diseases. Given the absence of effective vaccines and treatments, the high mortality rate, and the potential for a global pandemic outbreak, SFTS was designated as one of the top 10 priority infectious diseases by the WHO in 2018 (Li et al., 2021).

Similar to other bunyaviruses, SFTSV particles are spherical, measure 80–100 nm in diameter, and have a unit membrane envelope, from which protrude polypeptide spikes 5–10 nm long (Yu et al., 2011). The SFTSV genome comprises three segments: large (L), medium (M), and small (S), with lengths of 6368, 3378, and 1744 nucleotides, respectively. The L segment encodes the RNA-dependent RNA polymerase (RdRp), which is essential for RNA transcription and replication. The M segment contains a single open reading frame that codes for a 1073-amino acid precursor of the glycoprotein. The glycoprotein plays a crucial role in virus assembly, formation of virus particle, and attachment to new target cells. Notably, the S segment encodes the nucleoprotein (NP) and nonstructural protein (NSs) in the reverse direction. The nucleoprotein encapsidates and packages genomic RNA into ribonucleoprotein complexes to protect it from degradation by exogenous nucleases or immune systems in the host cell (Sun et al., 2012). The NP and NSs proteins play crucial roles in the replication of SFTSV.

Despite the broad geographical distribution of SFTSV isolates, they exhibit more than 90% sequence similarity (Liu et al., 2014). While several studies have addressed the classification of SFTSV, a standardized classification for this virus has yet to be established (Lam et al., 2013; Yoshikawa et al., 2015; Liu J. W. et al., 2016). Currently, two main genotyping methods are in use: one based on lineages A-F, and the other distinguishing Chinese and Japanese lineages. Therefore, more mutual understanding and discussion are required to establish a unified nomenclature for SFTSV genotypes. Recombination and reassortment are crucial evolutionary mechanisms for viruses, which might increase their virulence and pathogenicity. In the case of segmented-genome viruses, reassortment emerges as an exceptionally efficient force driving evolution, enabling viruses to adapt to new environments and even alter their host tropism (Kuiken et al., 2006). It has been reported that high rates of reassortment in segmented-genome viruses are responsible for genetic evolution, leading to the emergence of novel strains and genotypes, heightened pathogenicity, increased transmissibility among vectors and hosts, and can even the triggering of new outbreaks (Briese et al., 2006). In the case of SFTSV, the tick vector, *Haemaphysalis longicornis*, and the vertebrate reservoir hosts provide an environment for coinfection through homologous recombination and natural reassortment (Zhuang et al., 2018). Several studies have demonstrated that genetic diversity and rapid evolution of SFTSV are driven by gene mutation, natural reassortment, and homologous recombination (He and Ding, 2012; Shi et al., 2017; Wu et al., 2021). However, due to its relatively recent discovery, the molecular mechanism underlying the genetic diversity of SFTSV have not been fully elucidated. While previous studies have provided fundamental insights into the evolutionary patterns of SFTSV, the included sequences were predominantly from pre-2016, limiting their ability to reflect the current state of genetic and pathogenic diversity of SFTSV (He and Ding, 2012; Lam et al., 2013; Yoshikawa et al., 2015; Liu J. W. et al., 2016; Shi et al., 2017; Wu et al., 2021). Furthermore, despite phylogenetic analysis revealing close relationships among SFTSV strains sampled from different countries and hosts, molecular evidence supporting these relationships remain scarce.

To address this research gap, we aimed to unravel the present genetic diversity and evolution patterns of SFTSV by employing comprehensive bioinformatics methods and leveraging the latest sequences available from Genbank. Encouragingly, our study identified novel positive selection sites, recombination events, and reassortment strains. These findings provide compelling evidence supporting transmission of SFTSV across seas and between species.

## Materials and methods

### Sequence dataset

Full-length sequences of S, M, and L segments were retrieved from Genbank.^1^ Accessions lacking information on country of origin and date of isolation were excluded from further analysis. To minimize redundancy and avoid overrepresentation of genomic information, the three sequences dataset were uploaded to the CID-HIT-EST program and clustered using a similarity threshold of 99.8% (Fu et al., 2012). Finally, relevant information regarding the selected SFTSV strains, including host, collecting location, and collection date, was collected. All sequences were aligned using MAFFT (version 7.520) (Nakamura et al., 2018), and further manually edited using MEGA (version 11) (Kumar et al., 2018).

### Recombination and reassortment analysis

Two approaches were employed to identify potential recombination events. Firstly, the pairwise homoplasy index (PHI) test was conducted for each segment to detect the presence of recombination events, *p* value <0.05 was considered statistically significant indicator of recombination (Bruen et al., 2006). Secondly, potential recombinants were identified using the RDP4 software (Martin et al., 2010). Seven methods available in RDP4, namely RDP, 3Seq, GENECONV, SiScan, Chimaera, LARD, and MaxChi, were utilized. A recombination event was considered genuine if it was detected by at least three of the seven methods with a *p* value cutoff of 0.05 (He et al., 2020). Upon identification of recombinant sequences, they were excluded, and the process was repeated until no further recombination events were detected.

For the detection of potential genetic reassortments, background information such as collection date, strain name, collection site was extracted from the L, M, and S sequences datasets. If the background information for all three gene sequences (L, M and S) was identical, we determined that these sequences originated from the same viral strain and they formed a full-length genome of the virus. Conversely, if the genotypes inferred from the L, M and S gene segments differed, it was considered a potential reassortment strain. All recombination/reassortment sequences were excluded from the subsequent analysis.

### Phylogenetic analysis

The best-fit model of nucleotide substitution for each dataset was determined using ModelFinder based to the Bayesian information criterion (BIC). To construct maximum likelihood (ML) phylogenetic trees, IQ-TREE (version 2.0.3) was employed, with 1,000 ultrafast bootstrap replicates (Nguyen et al., 2015). Bayesian (BI) phylogenetic trees were reconstructed using Mrbayes (version 3.2.7) (Ronquist et al., 2012). Bayesian Markov chain Monte Carlo analysis was run for 50 million steps, 10% of which were removed as burn-in and sampled every 1,000 steps.

The pairwise genetic differences between each sequence and the earliest strain were calculated using MEGA (version 11) (Kumar et al., 2018). Subsequently, we employed the least squares method to fit a regression line relating genetic distance and time. Additionally, we conducted calculations for nucleotide pairwise genetic distances among all SFTSV sequences in each region using MEGA (version 11) (Kumar et al., 2018). We then compared the pairwise distances between two regions utilizing the Mann–Whitney *U*-test. All statistical analyses were performed using R software (version 4.2.2). *p* < 0.05 was considered statistically significant.

### Selection pressure analysis

Shannon entropy values were computed using the Shannon Entropy-One tool available at Los Alamos National Laboratory.^2^ The selection pressure exerted on the sequences was assessed by calculating the ratio of non-synonymous (*d_N_*) to synonymous (*d_S_*) nucleotide substitutions (*d_N_*/*d_S_*) per site through single likelihood ancestor counting (SLAC) using the Datamonkey server implementation of HyPhy (Delport et al., 2010). Codons were categorized as neutral (*d_N_*/*d_S_* = 1), undergoing positive selection (*d_N_*/*d_S_* > 1), or undergoing purifying selection (*d_N_*/*d_S_* < 1) (Suzuki and Gojobori, 1999). Positive selection sites were identified by SLAC, the fast unbiased Bayesian approximation (FUBAR), mixed effects model of evolution (MEME), and fixed-effects likelihood (FEL) (Suzuki and Gojobori, 1999). For SLAC, FEL, and MEME, only sites detected by at least two methods with statistically significant values (*p* value <0.05) were considered indicative of positive selection. FUBAR required a posterior probability ≥0.90 (Liu J. W. et al., 2016).

## Results

### Phylogenetic analysis of SFTSV

**Figure 1:**
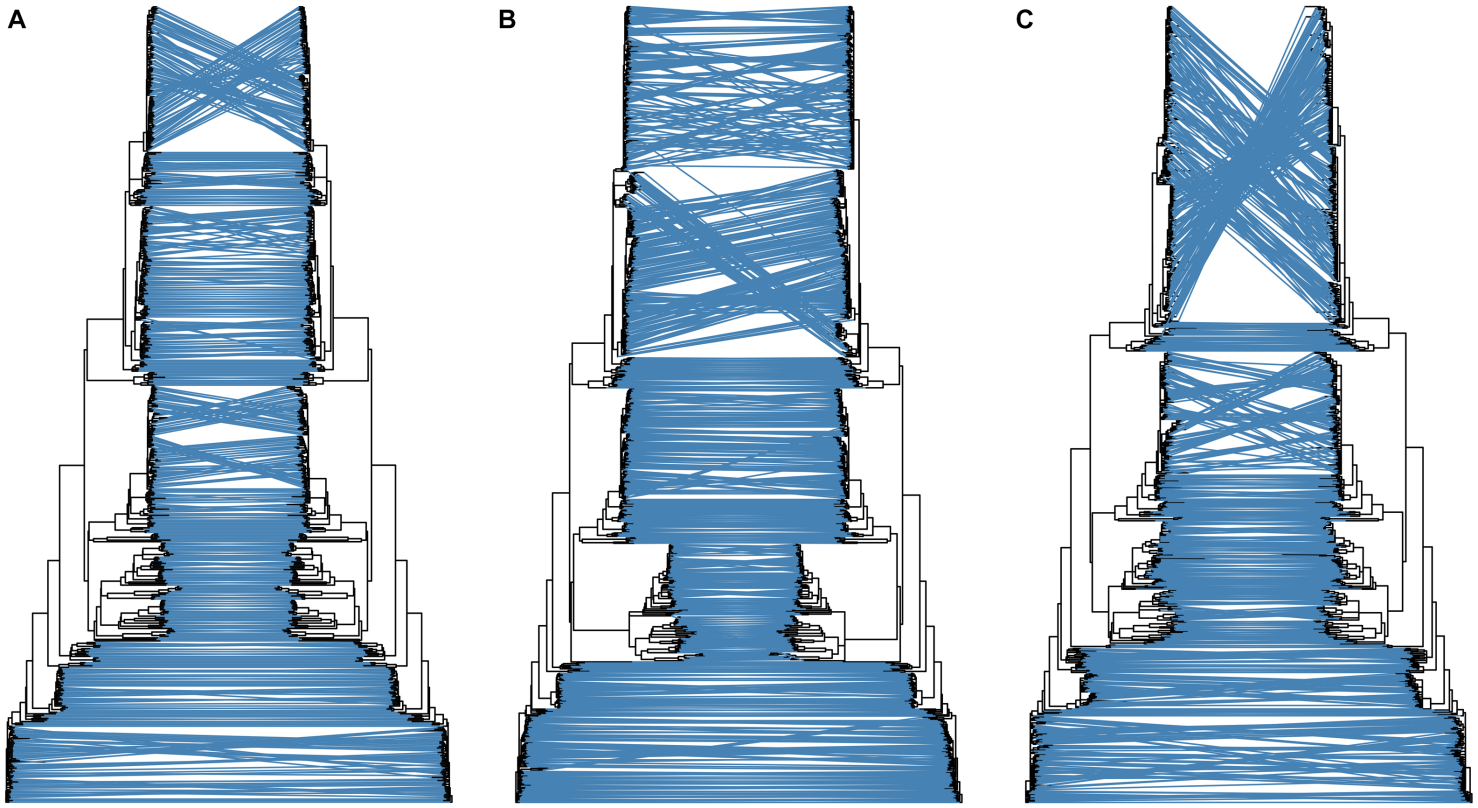
Comparison of topology of ML and BI trees for the L **(A)**, M **(B)**, and S **(C)** segments. For each subgraph, the left is ML tree and the right is BI tree.

**Figure 2:**
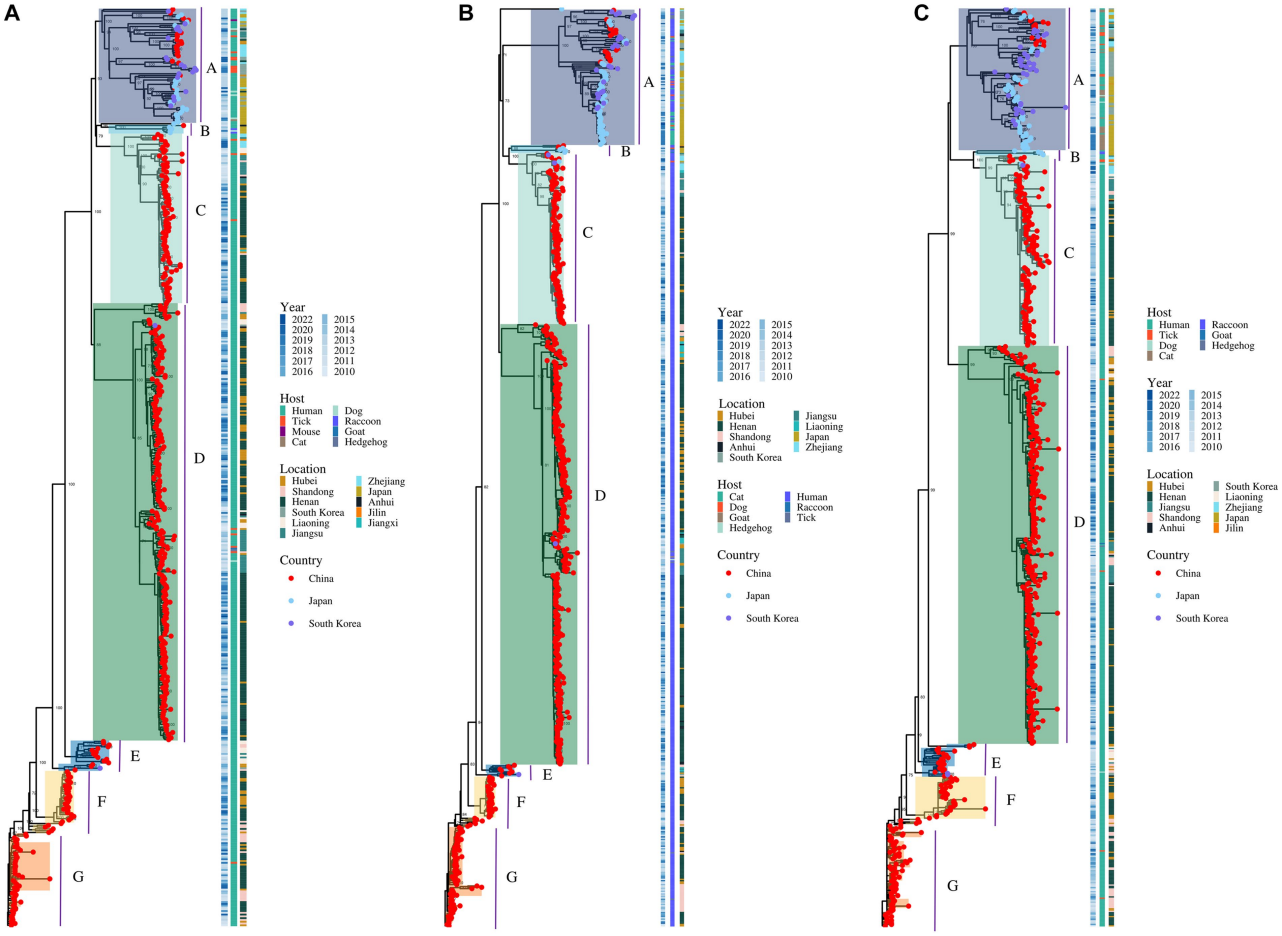
The ML trees of SFTSV genome for the L **(A)**, M **(B)**, and S **(C)** segments. The values of the bootstrap percentage (>70) are shown next to the branches.

**Figure 3:**
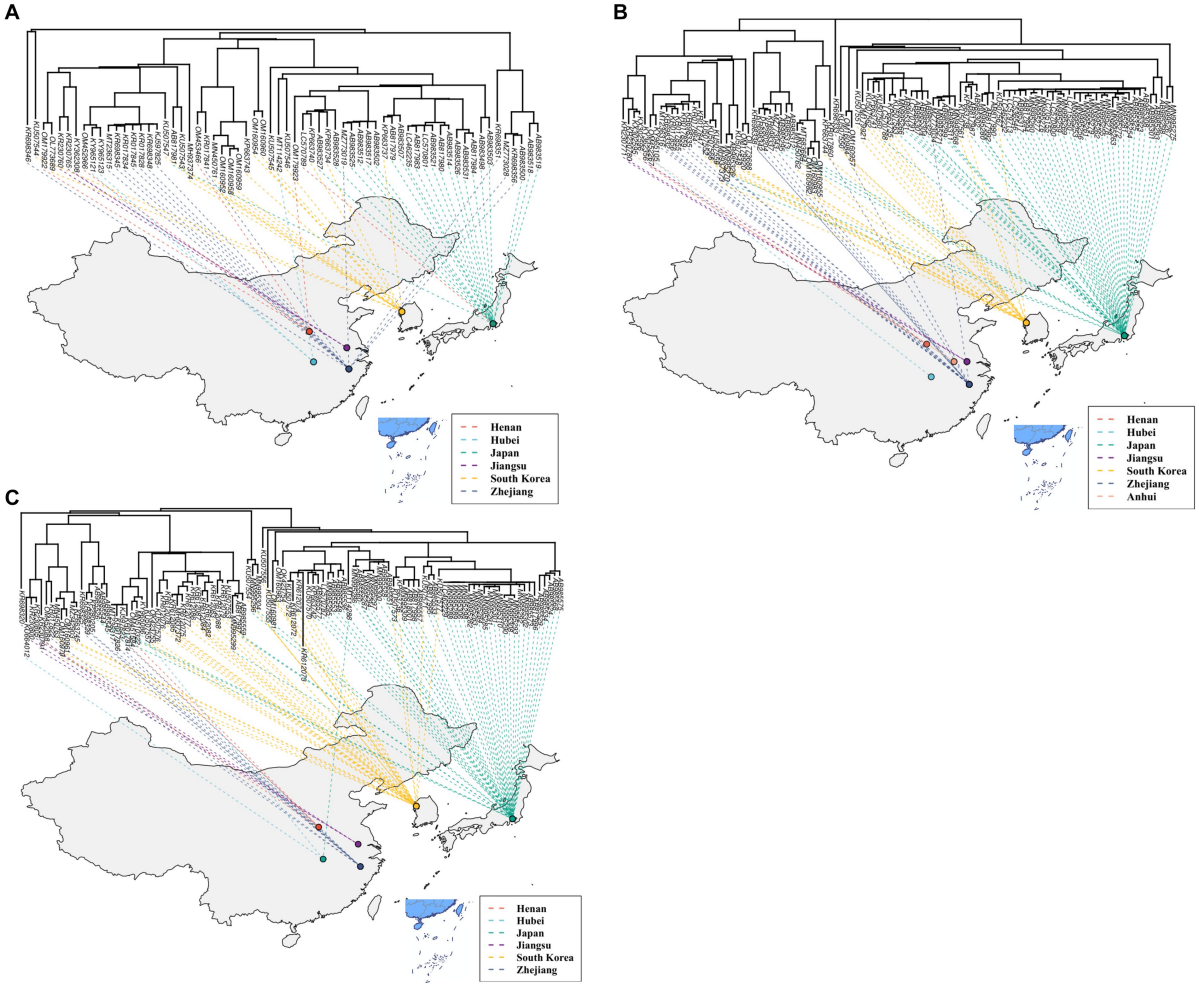
The geographical distribution of genotype A for the L **(A)**, M **(B)**, and S **(C)** segments.

**Figure 4:**
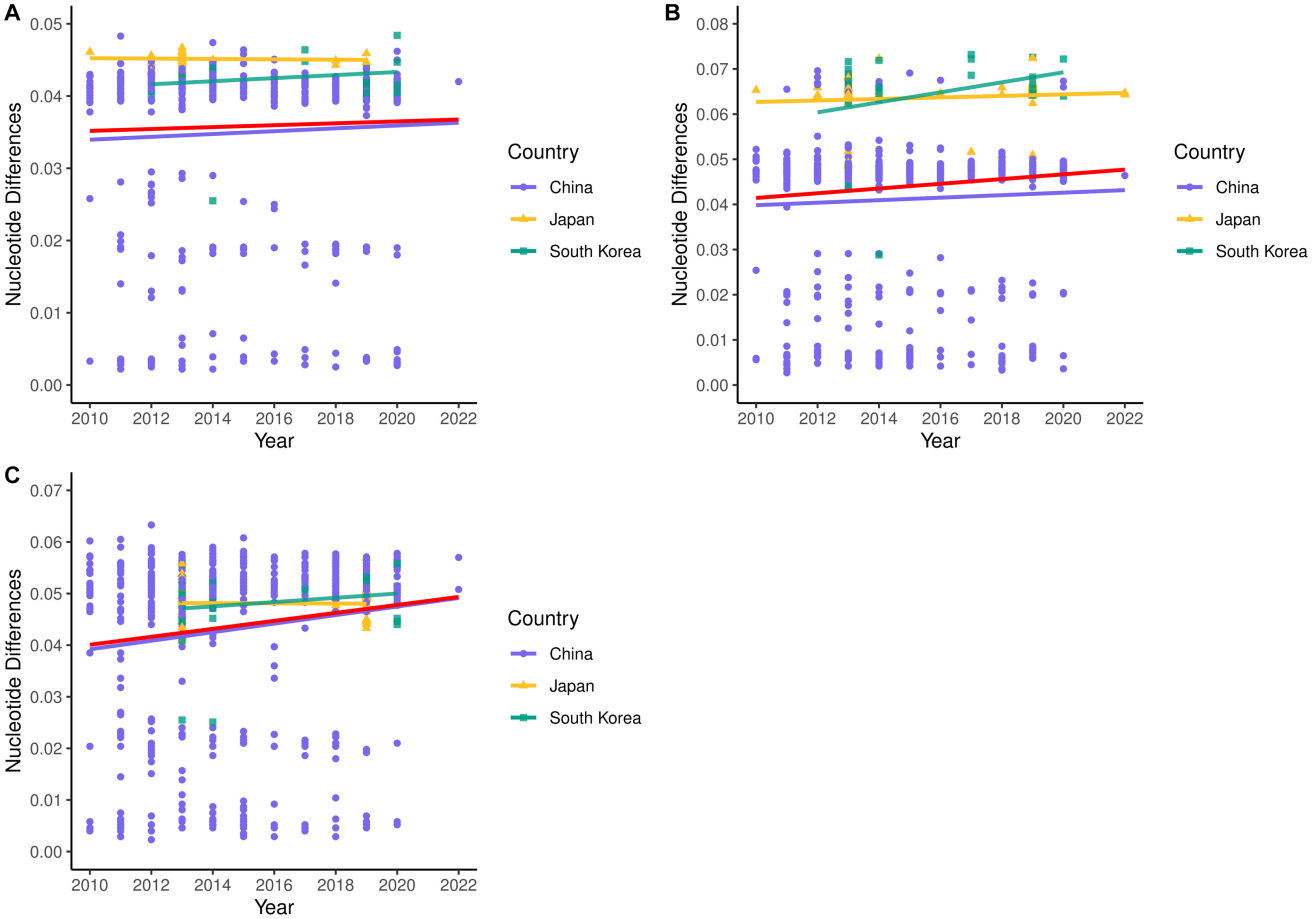
Nucleotide pairwise differences of each sequence compared to the earliest strain for L **(A)**, M **(B)**, and S **(C)** segments.

**Figure 5:**
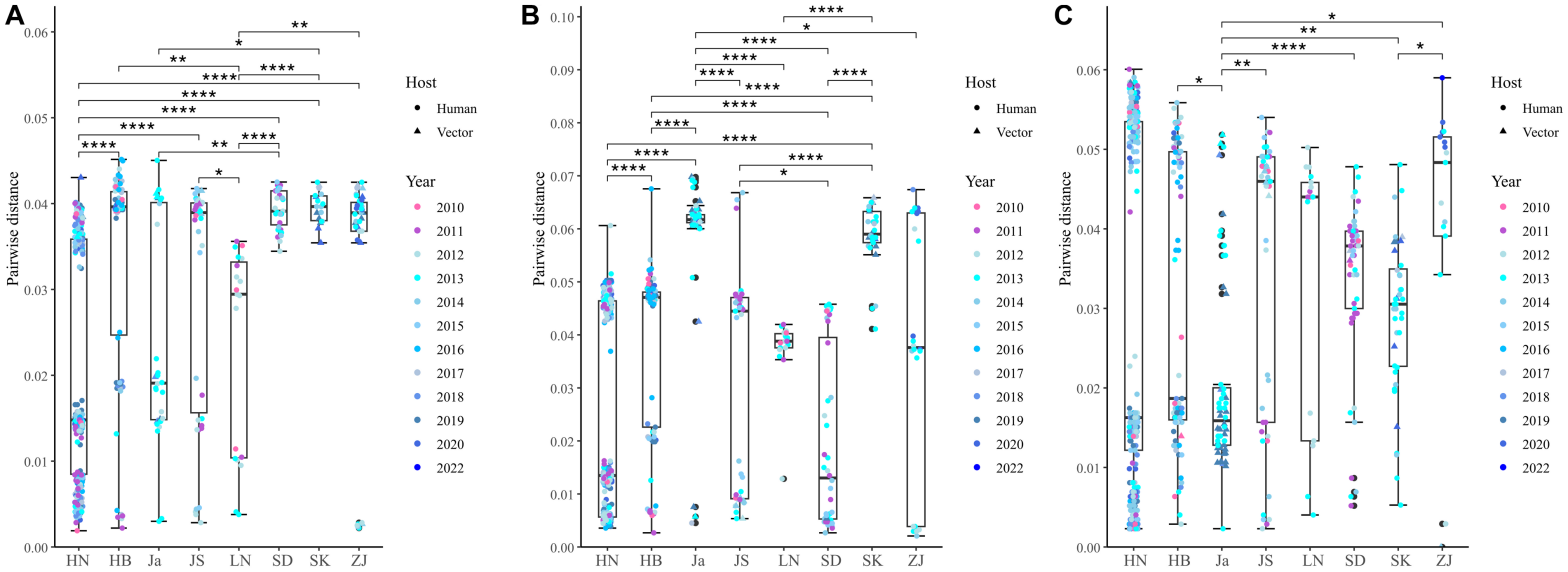
Nucleotide pairwise differences across different regions compared to the earliest strain for L **(A)**, M **(B)**, and S **(C)** segments. *p < 0.05; **p < 0.01; ***p < 0.001; ****p < 0.0001. HN, Henan; HB, Hubei; Ja, Japan; JS, Jiangsu; LN, Liaoning; SD, Shandong; SK, South Korea; ZJ, Zhejiang.

A total of 514 strains, 692 strains, and 699 strains were included in the phylogenetic analysis of the L, M, and S segments, using both maximum likelihood and Bayesian methods (Supplementary Table S1). Approximately 90% of the datasets consisted of strains from China and patients. The ML and BI trees exhibited similar topologies for each segment as well as among the three segments (Figure 1). Based on the ML tree, we classified genotypes, resulting in seven distinct genotypes (A, B, C, D, E, F, G) observed in the ML trees of the L, M and S segments (Figure 2). Genotypes C, D and G appeared to be the most common strains in mainland China. Genotype A primarily encompassed Japanese strains and was the only genotype that included strains sampled from China, Japan, and South Korea. Strains from South Korea formed their own distinct genotype, genotype B. A few strains from South Korea and Japan clustered closely with Chinese strains. The epidemiological information of the three segments is shown in Supplementary Figure S1. Shandong and Hubei presented diverse genotypes. The geographical distribution of genotype A is illustrated in Figure 3. Notably, Zhejiang Province in China contributed a larger number of strains to genotype A compared to other SFTSV epidemic regions in China. Genetic analyses did not reveal a significant accumulation of substitutions in the nucleotide sequences across the three segments, although the S segment exhibited a slightly higher substitution rate (Figure 4). We conducted further analysis of the pairwise genetic distance across regions (Figure 5). Overall, the pairwise genetic distance observed in the L segment was smaller compared to that of the M and S segments. The genetic distance varied between different segments within the same region, as well as between the same segment across different regions. In most regions, the pairwise genetic distance exhibited a distribution that was concentrated around both high and low values. Similarly, we did not observe any significant accumulation of pairwise genetic distance over time within the same segment and region. Specifically, for the L segment, the pairwise distance distribution of Shandong, South Korea, and Zhejiang was relatively similar, with a narrower range (Figure 5A). The genetic distance of the M segment showed similarity between Japan and South Korea (Figure 5B).

### Recombination and reassortment analysis of SFTSV

The PHI test results indicated a low likelihood of recombination within the S segment (*p* = 0.630) and a high probability of recombination within the L and M segments (*p* = 9.13E-10 and *p* = 0.0245, respectively). This suggests that homologous recombination within the L and M segments played a significant role in the evolution of SFTSV. 54, 36 and 11 potential recombination events in the L, M and S segments, respectively (Supplementary Tables S2–S4), and 41.58% (42/101) of these recombination events were characterized by recombinants and parents originating from distinct regions. Notably, in China, Zhejiang Province was the only province where major parents originated from other countries. Among the provinces included in our study, Henan Province, which had the largest number of strains, exhibited the highest number of detected recombination events in both the L and M segments, followed by Hubei Province. The recombination events detected in S segment were concentrated in Henan, Hubei, Shandong, Jiangsu and Zhejiang. Multiple recombination events occurred in some L (KC292336, KR698352, and OM453432) and M (KR698339) segments. Only one recombination event was detected in Japan. Furthermore, several recombination events involving humans and vectors (tick, sheep, and goat) were also detected. Among them, the strain (accession number: KT890281) isolated from a tick in Jilin exhibited the greatest distance compared to its parent strains, and two recombination events were supported by all seven detection methods.

**Figure 6:**
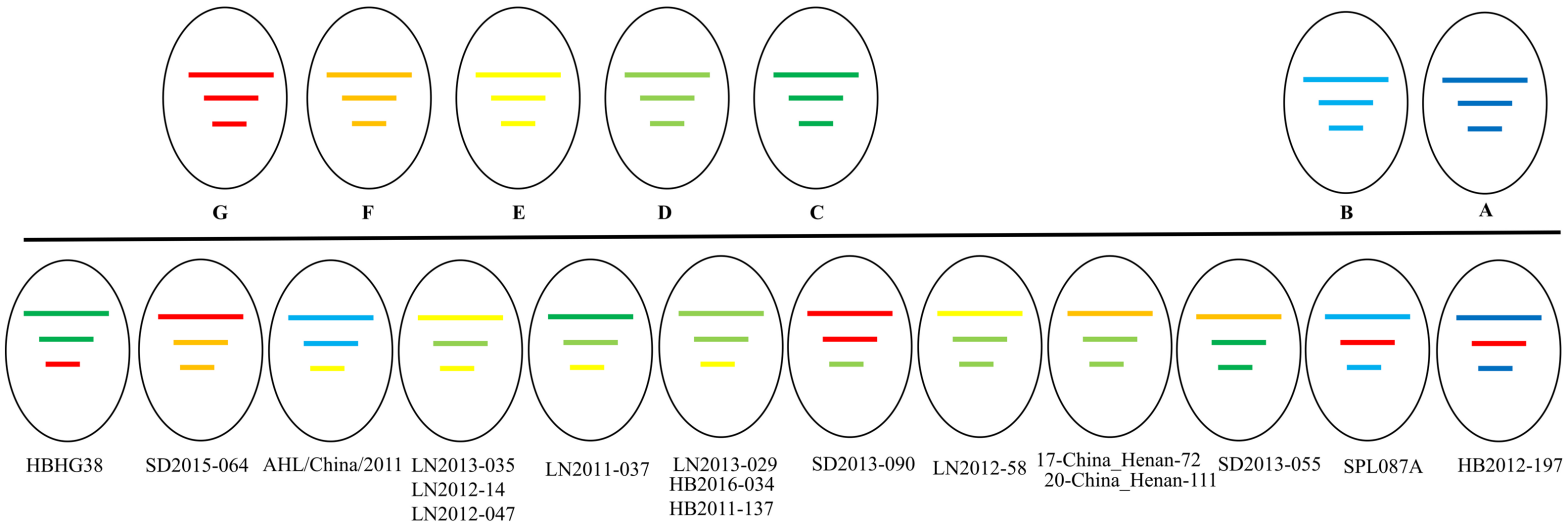
The genetic constellation of the potential SFTSV reassortments.

Our phylogenetic analyses revealed a total of 17 potential reassortments (Figure 6). The background information of these reassortment strains is summarized in Table 1. All reassortment strains were collected before 2018. Among the different regions examined, Liaoning had the highest number of reassortments, followed by Hubei and Shandong. Henan, which had a substantial number of strains and recombination events, exhibited two reassortments. Notably, the genotypes of the three segments in the LN2011-037 strain were distinct. Additionally, the reassortment strain identified in Japan belonging to different country clades.

**Table 1:** Positive selection sites using Datamonkey.

Gene	Model	Number of positive selection sites (Location of sites)	Number of purfing selection sites	Location of identified positive selection sites	*d_N_*/*d_S_*
Rdrp	FEL	3 (2, 691, 1353)	1364	2, 671, 1353	0.0561
	FUBAR	4 (2, 691, 1116, 1353)	1805		
	MEME	(2, 4, 68, 691, 1097, 1202, 1353, 1655, 1662, 1675, 1684, 1896, 1960, 2063)	–		
	SLAC	3 (2, 691, 1353)	1070		
Glycoprotein	FEL	3 (22, 298, 404)	566	22, 298, 404	0.108
	FUBAR	3 (22, 37, 298)	796		
	MEME	4 (13, 298, 404, 1018)	–		
	SLAC	1 (298)	433		
NP	FEL	–	146	–	0.0479
	FUBAR	–	188		
	MEME	–	–		
	SLAC	–	121		
NSs	FEL	2 (9, 289)	123	9, 289	0.1230
	FUBAR	2 (9, 281, 289)	172		
	MEME	2 (9, 289)	–		
	SLAC	1 (289)	86		

### Selection pressure on SFTSV

**Figure 7:**
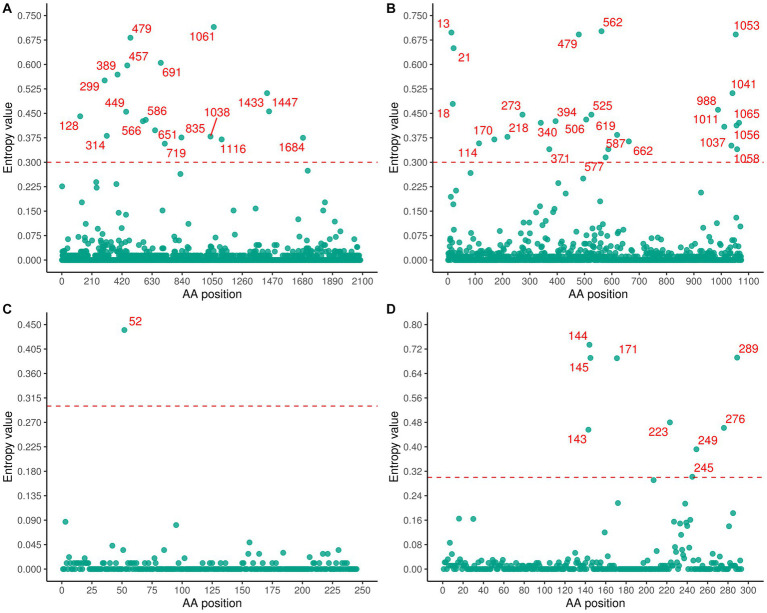
Shannon entropy was calculated to quantify amino acid variation for each site in RdRp **(A)**, glycoprotein **(B)**, NP **(C)**, and NSs **(D)**.

A significant number of variable residues were observed in RdRp and glycoprotein (Figure 7). In RdRp, the variable residues were predominantly located between positions 200 and 800 of the amino acid sequence (Figure 7A), while residues 700–1000 in the glycoprotein were conserved (Figure 7B). The variable residues of NSs occurred between positions 140 and 190 of the amino acid sequence (Figure 7D). Only one variable residue was detected in NP (Figure 7C). The *d_N_*/*d_S_* values for the RdRp, glycoprotein, NP, and NSs genes were 0.056, 0.108, 0.048, and 0.123, respectively, indicating that SFTSV experienced purifying selection (Table 2). Three sites (2, 671, 1353) in RdRp were identified as being under strong positive selection according to three methods (FEL, FUBAR, SLAC) (Table 2). In the glycoprotein, three positive selection sites (22, 298, 404) were identified, with two (22, 404) detected by two methods and the remaining site (298) detected by all methods (Table 2). Within the NSs gene, two sites (9, 289) were identified as positive selection sites (Table 2). No positive selection sites were found in NP gene.

**Table 2:** The background information of reassortment strains.

Strain name	Host	Collection time	Location
HBHG38	Patient	2017-07-28	Hubei, China
SD2015-064	Patient	2015-05-04	Shandong, China
AHL/China/2011	Patient	2011-06-13	Anhui, China
LN2013-035	Patient	2013-07-31	Liaoning, China
LN2012-14	Patient	2012-07-04	Liaoning, China
LN2012-047	Patient	2012-08-03	Liaoning, China
LN2011-037	Patient	2011-08-10	Liaoning, China
LN2013-029	Patient	2013-07-03	Liaoning, China
HB2016-034	Patient	2016-06-06	Hubei, China
HB2011-137	Patient	2011-09-29	Hubei, China
SD2013-090	Patient	2013-05-15	Shandong, China
LN2012-58	Patient	2012-08-06	Liaoning, China
17-China_Henan-72	Patient	2017	Henan, China
20-China_Henan-111	Patient	2020	Henan, China
SD2013-055	Patient	2013-06-23	Shandong, China
SPL087A	Patient	2013	Japan
HB2012-197	Patient	2012-07-30	Hubei, China

## Discussion

Due to the relatively recent identification of SFTSV, our understanding of the virus’s evolutionary patterns, phylogeny, and genetic variations remains incomplete, impeding the development of effective vaccines and antiviral treatments for SFTSV. In this study, we addressed this knowledge gap by leveraging a comprehensive dataset comprising SFTSV sequences from diverse geographic regions and spanning extended time periods.

Several published studies proposed nomenclature for SFTSV that divided the virus into two distinct clades based on the country of origin, such as C1 to C5 (Chinese clade) and J1 to J2 (Japanese clade) (Yoshikawa et al., 2015; Lv et al., 2017; Shi et al., 2017). However, our phylogenetic analysis revealed the existence of seven lineages, named A to G, based on full-length sequences from China, Japan, and South Korea (Lam et al., 2013; Liu J. W. et al., 2016). Remarkably, the tree patterns observed for the three segments exhibited significant similarities. Within the same sub-genotype, sequences isolated from the same locations were intermingled with those from different locations. Unlike previous study, strains sampled from different countries were clustered together, reflecting potential connections among strains from various regions (Yoshikawa et al., 2015; Lv et al., 2017). Research has demonstrated that viruses in China and South Korea were likely transmitted multiple times from Japan across the East China Sea and/or the Sea of Japan, and vice versa (Yoshikawa et al., 2015). In our study, no evidence of host adaptation was detected, and specific branches were not associated with isolation time or sample category, indicating the absence of geographical or species barriers. This may be attributed to the shared enzootic vectors and potential reservoirs (Yoshikawa et al., 2015; Chen et al., 2022). Consistent with previous findings (Liu L. et al., 2016), strains isolated from Japan, Zhejiang Province, and South Korea formed a sub-genotype, indicating close relationships between strains from China, Japan, and South Korea (Fu et al., 2016) reported that SFTSV genotype B strains were transmitted from South Korea to the Zhejiang Province and Japan, proposing that international travel and the migration of birds increased the possibility of SFTSV transmission across seas. Thus, conducting molecular epidemiological investigations of SFTSV focusing on Chinese coastal regions will not only provide insights into SFTSV evolution and transmission but also offer valuable information for the prevention and control of SFTS in East Asia. Notably, SFTSV displayed notable variations in evolutionary patterns among different regions and segments, without a linear accumulation of nucleotide substitutions within segments and regions. Thus, a wider geographic sampling of SFTSV is required to investigate the mechanisms underlying this divergent evolution.

Recombination plays a critical role in shaping the evolutionary genetics of RNA viruses (Moya et al., 2004). Recombination is considered a prominent genetic mechanism in segmented, positive-sense RNA viruses, as well as certain DNA viruses, such as hepatitis B virus (Shi et al., 2012). Conversely, it has been traditionally regarded as rare in negative-strand RNA viruses (Simon-Loriere and Holmes, 2011). Nevertheless, evidence of recombination has been discovered in the L and M segments of SFTSV, suggesting that recombination plays a potential role in the rapid evolution and generation of increased genetic diversity in SFTSV (He and Ding, 2012). Unfortunately, the number of investigated SFTSV strains was relatively small and the results had limited statistical support. In this study, based on updated data, a total of 101 recombination events were identified across all segments, providing new evidence for the significant role of recombination in the evolution of SFTSV. Notably, the recombination event observed in Zhejiang and Japan was supported by all seven test methods. The long-distance migration of SFTSV may be facilitated by ticks attaching to the hosts capable of long-distance mobility (Liu L. et al., 2016; Shi et al., 2017). However, the specific ways in which recombination may potentially affect the biological properties of these viruses warrant further in-depth investigation. Reassortment events occur when the virus has at least two segmented genomes. In the case of two or more segmented viruses infecting a single cell simultaneously, genomic segments have the potential to be packaged into progeny viruses randomly. Then, the progeny might inherit genomic segments from more than one parent, obtaining increased genetic variability. Therefore, reassortment is prevalent among viruses with segmented genomes (Simon-Loriere and Holmes, 2011), and reassortment can enhance pathogenicity and increase transmissibility among vectors and hosts (Kuiken et al., 2006). Seventeen SFTSV strains were identified as reassortments in our analysis, which were isolated from human samples, suggesting that reassortment events occurred frequently in SFTSV hosts. Liaoning Province, located in the northeast of China, exhibited the highest proportion of reassortment strains, with a unique stain consisting of three segments of different genotypes. Furthermore, our study provided updated information on reassortment and recombination events, including the identification of sea-crossing and species-crossing recombination events, which were discovered for the first time. These findings underscore the significance and necessity of monitoring the spread of SFTSV across regions and species. These new discoveries indicated that reassortment and recombination events could potentially drive the evolution of SFTSV, posing challenges for the development of SFTSV-targeted vaccines and the prevention and control of SFTS. However, further investigation is needed to understand how these events alter the biological properties of SFTSV.

Our findings indicated that purifying selection may be another factor influencing the evolution of SFTSV, consistent with previous studies (Liu J. W. et al., 2016; Liu L. et al., 2016). We observed low *d_N_*/*d_S_* values in all four coding genes, along with several sites showing positive selection. The relatively higher *d_N_*/*d_S_* value of glycoproteins, encoded by segment M, could be attributed to their accessibility to antibodies, although it is unclear if mutations in these sites can impact SFTSV virulence (Liu J. W. et al., 2016). The high *d_N_*/*d_S_* value of NSs may be a result of the co-evolutionary battle between host immunity and the virus (Lam et al., 2013). In summary, further studies employing reverse genetics are warranted to explore the relationship between mutations in these positively selected sites and the virulence of SFTSV.

In conclusion, our study investigated the evolution of SFTSV collected between 2010 and 2022, encompassing samples from China, Japan, and South Korea. Our analysis revealed the influence of selection pressure, recombination, and reassortment in shaping the genetic diversity of SFTSV. Notably, the identification of novel recombination and reassortment events provides compelling evidence supporting the transmission of SFTSV across seas and between different species. These findings contribute to our understanding of the genetic origins of SFTSV strains in humans and provide valuable insights into the molecular epidemiology, genetic diversity, and evolutionary patterns of SFTSV.

## Data availability statement

The original contributions presented in the study are included in the article/Supplementary material, further inquiries can be directed to the corresponding authors.

## Author contributions

YW, BP, ZK, and HW designed and implemented the study. ZW, XX, HL, and XC collected and managed the data. YW, BP, HL, and XT performed bioinformation analysis and prepared figures. YW, BP, and WM conceived the idea, interpreted the data, wrote, reviewed, and edited the manuscript. ZK and HW reviewed, edited, and revised the manuscript. All authors have read and approved the final manuscript.

## Funding

This work was financially supported by Natural Science Foundation of Shandong Province (ZR2022MH130) and Shandong Medical and Health Science and Technology Development Plan (No. 202012051274). The funder played no role in study design, data collection, and analysis, the decision to publish, or preparation of the manuscript.

## Conflict of interest

The authors declare that the research was conducted in the absence of any commercial or financial relationships that could be construed as a potential conflict of interest.

## Publisher’s note

All claims expressed in this article are solely those of the authors and do not necessarily represent those of their affiliated organizations, or those of the publisher, the editors and the reviewers. Any product that may be evaluated in this article, or claim that may be made by its manufacturer, is not guaranteed or endorsed by the publisher.

## Footnotes

^1^https://www.ncbi.nlm.nih.gov/genbank/, accessed on December 19, 2022.

^2^ https://www.hiv.lanl.gov/content/sequence/ENTROPY/entropy.html
