# Supplementary material for: Genomic diversity and evolution analysis of severe fever with thrombocytopenia syndrome in East Asia from 2010 to 2022
Source: Front Microbiol. 2023 Aug 21;14:1233693. doi: 10.3389/fmicb.2023.1233693 (PMC10476882; doi:10.3389/fmicb.2023.1233693)
Supplement: Supplementary file 1 [file Data_Sheet_1.zip › Supplementary Table S1.docx]

**Supplementary Table S1 The information of included sequences**

| L segment | | | | |  | M segment | | | | |  | S segment | | | | |
| --- | --- | --- | --- | --- | --- | --- | --- | --- | --- | --- | --- | --- | --- | --- | --- | --- |
| Accession | Host | Location | Year | Genotype |  | Accession | Host | Location | Year | Genotype |  | Accession | Host | Location | Year | Genotype |
| NC_018136 | Human | Hubei, China | 2010 | G |  | NC_018138 | Human | Hubei, China | 2010 | G |  | NC_018137 | Human | Hubei, China | 2010 | G |
| MT114237 | Human | Hubei, China | 2011 | G |  | OM452940 | Human | Henan, China | 2020 | G |  | OM451991 | Human | Henan, China | 2017 | G |
| MT005210 | Human | Shandong, China | 2011 | G |  | MT114251 | Human | Hubei, China | 2011 | G |  | OM451637 | Human | Henan, China | 2013 | G |
| KF711883 | Human | Henan, China | 2012 | G |  | OM452610 | Human | Henan, China | 2018 | G |  | OM451638 | Human | Henan, China | 2013 | G |
| MT005222 | Human | Shandong, China | 2013 | G |  | OM452245 | Human | Henan, China | 2011 | G |  | JQ317171 | Human | Jiangsu, China | 2010 | D |
| OM453467 | Human | Henan, China | 2020 | G |  | KF711920 | Human | Henan, China | 2012 | G |  | KF711891 | Human | Henan, China | 2012 | G |
| MT005225 | Human | Shandong, China | 2013 | G |  | OM452475 | Human | Henan, China | 2016 | G |  | KU738905 | Human | Hubei, China | 2014 | G |
| OM453289 | Human | Henan, China | 2018 | G |  | MN510162 | Human | Henan, China | 2018 | G |  | OM451809 | Human | Henan, China | 2015 | G |
| MT005204 | Human | Shandong, China | 2011 | G |  | OM452685 | Human | Henan, China | 2017 | G |  | OM451916 | Human | Henan, China | 2018 | G |
| MN509870 | Human | Henan, China | 2012 | G |  | MN510157 | Human | Henan, China | 2018 | G |  | OM451821 | Human | Henan, China | 2015 | G |
| MN509836 | Human | Henan, China | 2011 | G |  | OM452570 | Human | Henan, China | 2016 | G |  | OM451880 | Human | Henan, China | 2016 | G |
| OM453275 | Human | Henan, China | 2017 | G |  | MT005252 | Human | Shandong, China | 2011 | G |  | MT114312 | Human | Shandong, China | 2013 | G |
| OM453367 | Human | Henan, China | 2017 | G |  | KX641913 | Human | Shandong, China | 2015 | G |  | JQ733568 | Human | Hubei, China | 2011 | G |
| OM453619 | Human | Henan, China | 2020 | G |  | MT005264 | Human | Shandong, China | 2013 | G |  | MT114282 | Human | Hubei, China | 2012 | G |
| MT005212 | Human | Shandong, China | 2011 | G |  | KX641915 | Human | Shandong, China | 2015 | G |  | MT114315 | Human | Shandong, China | 2013 | G |
| MT005208 | Human | Shandong, China | 2011 | G |  | MT005250 | Human | Shandong, China | 2011 | G |  | MT114301 | Human | Shandong, China | 2011 | G |
| MT005215 | Human | Shandong, China | 2012 | G |  | KX641914 | Human | Shandong, China | 2015 | G |  | MT114302 | Human | Shandong, China | 2011 | G |
| MT005232 | Human | Shandong, China | 2014 | G |  | MT005277 | Human | Shandong, China | 2014 | G |  | MT114294 | Human | Shandong, China | 2011 | G |
| MT005205 | Human | Shandong, China | 2011 | G |  | MT005267 | Human | Shandong, China | 2013 | G |  | MT114325 | Human | Shandong, China | 2014 | G |
| MT005240 | Human | Shandong, China | 2015 | G |  | MT005271 | Human | Shandong, China | 2014 | G |  | KX641918 | Human | Shandong, China | 2015 | G |
| JQ733567 | Human | Hubei, China | 2011 | G |  | MT005243 | Human | Shandong, China | 2011 | G |  | MT114291 | Human | Shandong, China | 2011 | G |
| MN509955 | Human | Henan, China | 2015 | G |  | MT413433 | Tick | Shandong, China | 2018 | G |  | KX641919 | Human | Shandong, China | 2015 | G |
| OM453598 | Human | Henan, China | 2019 | G |  | MT005280 | Human | Shandong, China | 2015 | G |  | MT114330 | Human | Shandong, China | 2015 | G |
| OM453621 | Human | Henan, China | 2020 | G |  | MT005274 | Human | Shandong, China | 2014 | G |  | OM452214 | Human | Henan, China | 2019 | G |
| OM453529 | Human | Henan, China | 2020 | G |  | MT005266 | Human | Shandong, China | 2013 | G |  | MT413434 | Tick | Shandong, China | 2017 | G |
| OM453318 | Human | Henan, China | 2017 | G |  | JN258706 | Human | Shandong, China | 2010 | G |  | OM451615 | Human | Henan, China | 2013 | G |
| MN509850 | Human | Henan, China | 2012 | G |  | MT005281 | Human | Shandong, China | 2015 | G |  | OM451846 | Human | Henan, China | 2015 | G |
| OM453309 | Human | Henan, China | 2018 | G |  | MT005247 | Human | Shandong, China | 2011 | G |  | OM451746 | Human | Henan, China | 2014 | G |
| OM453007 | Human | Henan, China | 2013 | G |  | MT005278 | Human | Shandong, China | 2014 | G |  | OM451839 | Human | Henan, China | 2015 | G |
| HQ419226 | Human | Henan, China | 2010 | G |  | MT005248 | Human | Shandong, China | 2011 | G |  | OM451707 | Human | Henan, China | 2014 | G |
| MN509898 | Human | Henan, China | 2013 | G |  | MT005269 | Human | Shandong, China | 2013 | G |  | OM451815 | Human | Henan, China | 2015 | G |
| OM453161 | Human | Henan, China | 2016 | G |  | MT005282 | Human | Shandong, China | 2015 | G |  | OM451690 | Human | Henan, China | 2014 | G |
| KF711882 | Human | Henan, China | 2012 | G |  | JQ733560 | Human | Hubei, China | 2011 | G |  | OM451739 | Human | Henan, China | 2014 | G |
| OM453005 | Human | Henan, China | 2013 | G |  | JQ733566 | Human | Hubei, China | 2011 | G |  | OM451728 | Human | Henan, China | 2014 | G |
| MW721874 | Tick | Henan, China | 2019 | G |  | OM452913 | Human | Henan, China | 2019 | G |  | MT114328 | Human | Shandong, China | 2015 | G |
| MT114238 | Human | Hubei, China | 2012 | G |  | JF951394 | Human | Hubei, China | 2010 | G |  | MT114293 | Human | Shandong, China | 2011 | G |
| JQ733564 | Human | Hubei, China | 2011 | G |  | OM452673 | Human | Henan, China | 2018 | G |  | JN258705 | Human | Shandong, China | 2010 | G |
| MN509912 | Human | Henan, China | 2014 | G |  | OM452662 | Human | Henan, China | 2018 | G |  | MT114300 | Human | Shandong, China | 2011 | G |
| OM453592 | Human | Henan, China | 2019 | G |  | OM452483 | Human | Henan, China | 2016 | G |  | KF711892 | Human | Henan, China | 2012 | G |
| MN509859 | Human | Henan, China | 2012 | G |  | OM452430 | Human | Henan, China | 2014 | G |  | OM451790 | Human | Henan, China | 2016 | G |
| OM453498 | Human | Henan, China | 2020 | G |  | MN510095 | Human | Henan, China | 2015 | G |  | MT114298 | Human | Shandong, China | 2011 | G |
| OM453620 | Human | Henan, China | 2020 | G |  | OM452308 | Human | Henan, China | 2013 | G |  | KP339897 | Human | Henan, China | 2014 | G |
| MT005236 | Human | Shandong, China | 2014 | G |  | KF711921 | Human | Henan, China | 2012 | G |  | KF917447 | Human | Henan, China | 2013 | G |
| KY965111 | Human | Hubei, China | 2016 | G |  | KF356540 | Human | Henan, China | 2012 | G |  | MT114322 | Human | Shandong, China | 2014 | G |
| OM453205 | Human | Henan, China | 2015 | G |  | OM452394 | Human | Henan, China | 2014 | G |  | MT114314 | Human | Shandong, China | 2013 | G |
| OM453543 | Human | Henan, China | 2019 | G |  | MN510066 | Human | Henan, China | 2013 | G |  | OM451903 | Human | Henan, China | 2017 | G |
| MT320814 | Human | Hubei, China | 2017 | G |  | MN510041 | Human | Henan, China | 2012 | G |  | OM451740 | Human | Henan, China | 2014 | G |
| MT005238 | Human | Shandong, China | 2015 | G |  | OM452336 | Human | Henan, China | 2013 | G |  | OM451943 | Human | Henan, China | 2018 | G |
| MT005203 | Human | Shandong, China | 2011 | G |  | OM452746 | Human | Henan, China | 2019 | G |  | OM451935 | Human | Henan, China | 2018 | G |
| MT005211 | Human | Shandong, China | 2011 | G |  | OM452636 | Human | Henan, China | 2018 | G |  | OM451841 | Human | Henan, China | 2016 | G |
| MT005237 | Human | Shandong, China | 2015 | G |  | OM452431 | Human | Henan, China | 2014 | G |  | OM451797 | Human | Henan, China | 2015 | G |
| MT005227 | Human | Shandong, China | 2013 | G |  | MN510042 | Human | Henan, China | 2012 | G |  | MT320801 | Human | Hubei, China | 2017 | G |
| MT114244 | Human | Hubei, China | 2013 | F |  | MN510032 | Human | Henan, China | 2012 | G |  | KY965076 | Human | Hubei, China | 2016 | G |
| MT005228 | Human | Shandong, China | 2013 | F |  | OM452417 | Human | Henan, China | 2014 | G |  | OM451576 | Human | Henan, China | 2012 | G |
| MT005214 | Human | Shandong, China | 2012 | F |  | MT114252 | Human | Hubei, China | 2012 | G |  | OM452053 | Human | Henan, China | 2019 | G |
| MN510013 | Human | Henan, China | 2018 | F |  | KX302598 | Human | Anhui, China | 2015 | G |  | OM452239 | Human | Henan, China | 2019 | G |
| MT005207 | Human | Shandong, China | 2011 | F |  | MW721876 | Tick | Shandong, China | 2019 | G |  | MT114276 | Human | Hubei, China | 2011 | G |
| MN509979 | Human | Henan, China | 2017 | F |  | JQ733563 | Human | Hubei, China | 2011 | G |  | MW721878 | Tick | Henan, China | 2019 | G |
| MT005219 | Human | Shandong, China | 2013 | F |  | KY965106 | Human | Hubei, China | 2016 | G |  | OM452094 | Human | Henan, China | 2020 | G |
| OM453256 | Human | Henan, China | 2016 | F |  | MT114246 | Human | Hubei, China | 2011 | G |  | KF917448 | Human | Henan, China | 2013 | G |
| KF356548 | Human | Henan, China | 2011 | F |  | OM452919 | Human | Henan, China | 2019 | G |  | KP339916 | Human | Henan, China | 2014 | G |
| KF711884 | Human | Henan, China | 2011 | F |  | MN510104 | Human | Hubei, China | 2015 | G |  | OM451563 | Human | Henan, China | 2012 | G |
| MN509998 | Human | Henan, China | 2018 | F |  | OM452437 | Human | Henan, China | 2014 | G |  | OM451658 | Human | Henan, China | 2013 | G |
| OM453451 | Human | Henan, China | 2020 | F |  | KF711919 | Human | Henan, China | 2012 | G |  | OM451831 | Human | Henan, China | 2015 | G |
| OM453029 | Human | Henan, China | 2013 | F |  | MN510101 | Human | Henan, China | 2015 | G |  | OM452243 | Human | Henan, China | 2020 | G |
| OM453514 | Human | Henan, China | 2020 | F |  | MN510163 | Human | Henan, China | 2018 | G |  | MT114305 | Human | Shandong, China | 2012 | G |
| MN510001 | Human | Henan, China | 2018 | F |  | MN510055 | Human | Henan, China | 2013 | G |  | HQ419239 | Human | Henan, China | 2010 | G |
| OM453552 | Human | Hubei, China | 2019 | F |  | MN510029 | Human | Henan, China | 2012 | G |  | MT114295 | Human | Shandong, China | 2011 | G |
| OM453614 | Human | Hubei, China | 2019 | F |  | OM452942 | Human | Henan, China | 2020 | G |  | KP339891 | Human | Henan, China | 2014 | G |
| KC292327 | Human | Henan, China | 2011 | F |  | MN510027 | Human | Henan, China | 2012 | G |  | OM452039 | Human | Henan, China | 2018 | G |
| OM453293 | Human | Hubei, China | 2018 | F |  | OM452752 | Human | Henan, China | 2019 | G |  | OM451662 | Human | Henan, China | 2013 | G |
| MN509915 | Human | Hubei, China | 2014 | F |  | OM452934 | Human | Henan, China | 2019 | G |  | OM451654 | Human | Henan, China | 2013 | G |
| OM453434 | Human | Hubei, China | 2019 | F |  | OM452528 | Human | Henan, China | 2015 | G |  | OM451575 | Human | Henan, China | 2012 | G |
| OM453327 | Human | Hubei, China | 2018 | F |  | OM452864 | Human | Henan, China | 2019 | G |  | MT114326 | Human | Shandong, China | 2014 | G |
| OM453274 | Human | Hubei, China | 2017 | F |  | MT320788 | Human | Hubei, China | 2017 | G |  | MT114316 | Human | Shandong, China | 2013 | G |
| KY440777 | Human | Hubei, China | 2015 | F |  | MT005279 | Human | Shandong, China | 2015 | F |  | MT114296 | Human | Shandong, China | 2011 | G |
| MN509939 | Human | Henan, China | 2015 | F |  | MT114258 | Human | Hubei, China | 2013 | F |  | HM802205 | Human | Shandong, China | 2010 | G |
| OM453277 | Human | Henan, China | 2017 | F |  | MT005256 | Human | Shandong, China | 2012 | F |  | MT114327 | Human | Shandong, China | 2015 | F |
| MN509999 | Human | Henan, China | 2018 | F |  | MT005270 | Human | Shandong, China | 2013 | F |  | MT114288 | Human | Hubei, China | 2013 | F |
| MN509917 | Human | Henan, China | 2014 | F |  | MN510127 | Human | Henan, China | 2016 | F |  | MT114318 | Human | Shandong, China | 2013 | F |
| OM452991 | Human | Henan, China | 2013 | F |  | MT005249 | Human | Shandong, China | 2011 | F |  | MT114304 | Human | Shandong, China | 2012 | F |
| OM453597 | Human | Hubei, China | 2019 | F |  | MN510144 | Human | Henan, China | 2017 | F |  | MT114297 | Human | Shandong, China | 2011 | F |
| OM453406 | Human | Hubei, China | 2018 | F |  | KF711925 | Human | Henan, China | 2011 | F |  | OM451952 | Human | Hubei, China | 2018 | F |
| KC292347 | Human | Henan, China | 2011 | F |  | KF711926 | Human | Henan, China | 2011 | F |  | OM452029 | Human | Hubei, China | 2018 | F |
| MN509933 | Human | Henan, China | 2015 | F |  | KF711923 | Human | Henan, China | 2011 | F |  | OM451920 | Human | Hubei, China | 2018 | F |
| MN509918 | Human | Henan, China | 2014 | F |  | KF356536 | Human | Henan, China | 2011 | F |  | OM451905 | Human | Henan, China | 2017 | F |
| OM453334 | Human | Henan, China | 2018 | F |  | OM452580 | Human | Henan, China | 2016 | F |  | OM451906 | Human | Henan, China | 2017 | F |
| MT005216 | Human | Shandong, China | 2012 | E |  | OM452708 | Human | Henan, China | 2018 | F |  | OM451747 | Human | Henan, China | 2014 | F |
| KP663731 | Human | South Korea | 2014 | E |  | MN510058 | Human | Henan, China | 2013 | F |  | OM451907 | Human | Henan, China | 2017 | F |
| KY965113 | Human | Hubei, China | 2016 | E |  | OM452835 | Human | Henan, China | 2020 | F |  | KF711898 | Human | Henan, China | 2011 | F |
| KY965109 | Human | Hubei, China | 2016 | E |  | OM452676 | Human | Henan, China | 2018 | F |  | OM451888 | Human | Henan, China | 2016 | F |
| KF887443 | Human | Liaoning, China | 2012 | E |  | MN510150 | Human | Hubei, China | 2017 | F |  | OM452014 | Human | Henan, China | 2018 | F |
| MT114213 | Human | Liaoning, China | 2013 | E |  | OM452650 | Human | Henan, China | 2018 | F |  | OM451734 | Human | Henan, China | 2014 | F |
| MT114204 | Human | Liaoning, China | 2011 | E |  | KC292321 | Human | Henan, China | 2012 | F |  | OM451611 | Human | Henan, China | 2013 | F |
| KC505135 | Human | Jiangsu, China | 2012 | E |  | OM452440 | Human | Henan, China | 2014 | F |  | OM451650 | Human | Henan, China | 2013 | F |
| KY362317 | Human | Jiangsu, China | 2015 | E |  | OM452589 | Human | Henan, China | 2016 | F |  | KF356525 | Human | Henan, China | 2011 | F |
| MT114201 | Human | Liaoning, China | 2010 | E |  | MN510087 | Human | Henan, China | 2015 | F |  | KF356524 | Human | Henan, China | 2011 | F |
| KF887441 | Human | Liaoning, China | 2012 | E |  | MN510146 | Human | Henan, China | 2017 | F |  | OM452174 | Human | Hubei, China | 2019 | F |
| KF887442 | Human | Liaoning, China | 2012 | E |  | MN510145 | Human | Henan, China | 2017 | F |  | OM452110 | Human | Hubei, China | 2020 | F |
| MT114208 | Human | Liaoning, China | 2012 | E |  | OM452406 | Human | Henan, China | 2014 | F |  | JQ317174 | Human | Jiangsu, China | 2010 | D |
| KF887445 | Human | Liaoning, China | 2012 | E |  | KY440776 | Human | Hubei, China | 2015 | F |  | MN510279 | Human | Hubei, China | 2017 | F |
| MT114207 | Human | Liaoning, China | 2012 | E |  | MN510103 | Human | Hubei, China | 2015 | F |  | KY440769 | Human | Hubei, China | 2016 | F |
| MT005234 | Human | Shandong, China | 2014 | E |  | OM452873 | Human | Hubei, China | 2019 | F |  | OM452219 | Human | Hubei, China | 2019 | F |
| MT005221 | Human | Shandong, China | 2013 | E |  | OM452739 | Human | Henan, China | 2018 | F |  | OM452031 | Human | Henan, China | 2018 | F |
| KU507544 | Human | South Korea | 2013 | A |  | OM452809 | Human | Hubei, China | 2020 | F |  | JQ317177 | Human | Jiangsu, China | 2010 | D |
| KR698346 | Human | Zhejiang, China | 2013 | A |  | OM452918 | Human | Hubei, China | 2019 | F |  | OM451959 | Human | Henan, China | 2018 | F |
| KR698351 | Human | Zhejiang, China | 2013 | A |  | OM452643 | Human | Hubei, China | 2018 | F |  | OM451787 | Human | Henan, China | 2015 | F |
| AB983519 | Human | Japan | 2013 | A |  | OM452425 | Human | Hubei, China | 2014 | F |  | OM452056 | Human | Hubei, China | 2019 | F |
| AB983518 | Human | Japan | 2013 | A |  | OM452755 | Human | Hubei, China | 2019 | F |  | OM451717 | Human | Henan, China | 2014 | F |
| MZ773028 | Human | Zhejiang, China | 2020 | A |  | KC292300 | Human | Henan, China | 2012 | F |  | OM451735 | Human | Hubei, China | 2014 | F |
| AB983500 | Human | Japan | 2013 | A |  | MN510052 | Human | Hubei, China | 2012 | F |  | OM451749 | Human | Henan, China | 2014 | F |
| KR698356 | Mouse | Zhejiang, China | 2014 | A |  | MN510071 | Human | Hubei, China | 2013 | F |  | OM451829 | Human | Henan, China | 2015 | F |
| MH937374 | Human | South Korea | 2017 | A |  | OM452935 | Human | Hubei, China | 2019 | F |  | OM451823 | Human | Hubei, China | 2015 | F |
| KU507543 | Human | South Korea | 2013 | A |  | MT005258 | Human | Shandong, China | 2012 | E |  | OM451813 | Human | Henan, China | 2015 | F |
| AB817981 | Human | Japan | 2012 | A |  | KY965092 | Human | Hubei, China | 2016 | E |  | JQ670932 | Human | Anhui, China | 2011 | E |
| OL773689 | Cat | South Korea | 2020 | A |  | KP663732 | Human | South Korea | 2014 | E |  | KP663733 | Human | South Korea | 2014 | E |
| OM179922 | Dog | Henan, China | 2019 | A |  | KC505136 | Human | Jiangsu, China | 2012 | E |  | KU507568 | Human | South Korea | 2013 | E |
| KY362308 | Human | Jiangsu, China | 2015 | A |  | KY362365 | Human | Jiangsu, China | 2015 | E |  | JQ693003 | Dog | Shandong, China | 2011 | E |
| KR230765 | Tick | Jiangsu, China | 2014 | A |  | MT114217 | Human | Liaoning, China | 2010 | E |  | KY965078 | Human | Hubei, China | 2016 | E |
| KR230760 | Human | Jiangsu, China | 2014 | A |  | MT114223 | Human | Liaoning, China | 2012 | E |  | JQ693013 | Human | Shandong, China | 2011 | E |
| KU507547 | Human | South Korea | 2013 | A |  | MT005276 | Human | Shandong, China | 2014 | E |  | KY965074 | Human | Hubei, China | 2016 | E |
| OM452966 | Human | Henan, China | 2012 | A |  | MT005263 | Human | Shandong, China | 2013 | E |  | MT114306 | Human | Shandong, China | 2012 | E |
| KY965123 | Human | Hubei, China | 2016 | A |  | AB985309 | Human | Japan | 2013 | G |  | MT114324 | Human | Shandong, China | 2014 | E |
| KY965121 | Human | Hubei, China | 2016 | A |  | KR017860 | Human | Zhejiang, China | 2012 | A |  | MT114311 | Human | Shandong, China | 2013 | E |
| KJ597825 | Human | Zhejiang, China | 2011 | A |  | KP663744 | Human | South Korea | 2014 | A |  | KC505137 | Human | Jiangsu, China | 2012 | E |
| KR698348 | Human | Zhejiang, China | 2013 | A |  | MT683684 | Human | South Korea | 2017 | A |  | MT114273 | Human | Liaoning, China | 2013 | E |
| KR017828 | Human | Zhejiang, China | 2012 | A |  | MN450762 | Tick | South Korea | 2017 | A |  | MT114263 | Human | Liaoning, China | 2011 | E |
| KR017845 | Human | Zhejiang, China | 2014 | A |  | OM160955 | Tick | South Korea | 2019 | A |  | KU664007 | Human | Hubei, China | 2012 | E |
| MT236315 | Tick | Zhejiang, China | 2014 | A |  | OM160983 | Tick | South Korea | 2019 | A |  | KF887431 | Human | Liaoning, China | 2012 | E |
| KR017834 | Human | Zhejiang, China | 2012 | A |  | OM160982 | Tick | South Korea | 2019 | A |  | KF887432 | Human | Liaoning, China | 2012 | E |
| KR698345 | Human | Zhejiang, China | 2013 | A |  | KR698338 | Human | Zhejiang, China | 2013 | A |  | MT114268 | Human | Liaoning, China | 2012 | E |
| OM160960 | Tick | South Korea | 2020 | A |  | AB985312 | Human | Japan | 2013 | A |  | KF887434 | Human | Liaoning, China | 2012 | E |
| OM160954 | Tick | South Korea | 2020 | A |  | AB985313 | Human | Japan | 2013 | A |  | MT114267 | Human | Liaoning, China | 2012 | E |
| KR017841 | Human | Zhejiang, China | 2012 | A |  | AB985295 | Human | Japan | 2013 | A |  | JQ693004 | Human | Shandong, China | 2011 | E |
| OM453466 | Human | Henan, China | 2020 | A |  | MZ773043 | Human | Zhejiang, China | 2020 | A |  | KY362341 | Human | Jiangsu, China | 2015 | E |
| KP663743 | Human | South Korea | 2014 | A |  | KR698342 | Human | Zhejiang, China | 2014 | A |  | MT114261 | Human | Liaoning, China | 2010 | E |
| MN450761 | Tick | South Korea | 2017 | A |  | KR698337 | Human | Zhejiang, China | 2013 | A |  | MT114271 | Human | Liaoning, China | 2013 | E |
| OM160952 | Tick | South Korea | 2019 | A |  | KP280204 | Human | Zhejiang, China | 2013 | A |  | KY965081 | Human | Hubei, China | 2016 | E |
| OM160959 | Tick | South Korea | 2019 | A |  | KY362356 | Human | Jiangsu, China | 2015 | A |  | MT114278 | Human | Hubei, China | 2011 | E |
| OM160958 | Tick | South Korea | 2019 | A |  | KR230771 | Human | Jiangsu, China | 2011 | A |  | KU507554 | Human | South Korea | 2013 | A |
| KU507545 | Human | South Korea | 2013 | A |  | KR230780 | Human | Jiangsu, China | 2014 | A |  | JQ317180 | Human | Jiangsu, China | 2011 | D |
| KU507546 | Human | South Korea | 2013 | A |  | KX302596 | Human | Anhui, China | 2014 | A |  | KC292273 | Human | Henan, China | 2012 | F |
| MT114242 | Human | Hubei, China | 2012 | A |  | KX302595 | Human | Anhui, China | 2014 | A |  | KR698320 | Human | Zhejiang, China | 2013 | A |
| AB983503 | Human | Japan | 2013 | A |  | OL773688 | Cat | South Korea | 2020 | A |  | KR698325 | Human | Zhejiang, China | 2013 | A |
| AB983498 | Human | Japan | 2013 | A |  | OM179920 | Dog | South Korea | 2019 | A |  | AB985545 | Human | Japan | 2013 | A |
| AB983502 | Human | Japan | 2013 | A |  | KU507548 | Human | South Korea | 2013 | A |  | AB985544 | Human | Japan | 2013 | A |
| AB983517 | Human | Japan | 2013 | A |  | AB985326 | Human | Japan | 2014 | A |  | AB985526 | Human | Japan | 2013 | A |
| AB983512 | Human | Japan | 2013 | A |  | MN995270 | Cat | Japan | 2019 | A |  | KP280205 | Human | Zhejiang, China | 2013 | A |
| AB983525 | Human | Japan | 2013 | A |  | MH937373 | Human | South Korea | 2017 | A |  | KR698329 | Human | Zhejiang, China | 2014 | A |
| MZ773019 | Human | Zhejiang, China | 2020 | A |  | KU507559 | Human | South Korea | 2013 | A |  | KR230791 | Human | Jiangsu, China | 2011 | A |
| OM179923 | Dog | Henan, China | 2020 | A |  | KU507566 | Human | South Korea | 2013 | A |  | KU664012 | Human | Hubei, China | 2012 | A |
| AB983528 | Human | Japan | 2012 | A |  | KU507552 | Human | South Korea | 2013 | A |  | KR230805 | Tick | Jiangsu, China | 2014 | A |
| KP663734 | Human | South Korea | 2014 | A |  | KY965105 | Human | Hubei, China | 2016 | A |  | KR230800 | Human | Jiangsu, China | 2014 | A |
| AB983527 | Human | Japan | 2013 | A |  | OM452267 | Human | Henan, China | 2012 | A |  | KR017822 | Human | Zhejiang, China | 2012 | A |
| KP663740 | Human | South Korea | 2014 | A |  | KR017847 | Human | Zhejiang, China | 2012 | A |  | OM452088 | Human | Henan, China | 2020 | A |
| LC570789 | Dog | Japan | 2018 | A |  | KR017864 | Human | Zhejiang, China | 2014 | A |  | KP663745 | Human | South Korea | 2014 | A |
| AB983507 | Human | Japan | 2013 | A |  | KR698335 | Human | Zhejiang, China | 2013 | A |  | KC292279 | Human | Henan, China | 2012 | D |
| KP663737 | Human | South Korea | 2014 | A |  | KR017859 | Human | Zhejiang, China | 2012 | A |  | MN450763 | Tick | South Korea | 2017 | A |
| AB817979 | Human | Japan | 2012 | A |  | MT236316 | Tick | Zhejiang, China | 2014 | A |  | KC292282 | Human | Henan, China | 2012 | D |
| AB983526 | Human | Japan | 2013 | A |  | KR698332 | Human | Zhejiang, China | 2013 | A |  | KC292283 | Human | Henan, China | 2012 | D |
| AB817984 | Human | Japan | 2010 | A |  | KR017855 | Human | Zhejiang, China | 2012 | A |  | KU507557 | Human | South Korea | 2013 | A |
| AB983531 | Human | Japan | 2013 | A |  | KR017856 | Human | Zhejiang, China | 2012 | A |  | KY965086 | Human | Hubei, China | 2016 | A |
| AB983514 | Human | Japan | 2013 | A |  | KR698333 | Human | Zhejiang, China | 2013 | A |  | MN510322 | Human | Henan, China | 2018 | A |
| AB817980 | Human | Japan | 2012 | A |  | MN995275 | Cat | Japan | 2019 | A |  | OM451584 | Human | Henan, China | 2012 | A |
| AB983521 | Human | Japan | 2013 | A |  | MN995267 | Cat | Japan | 2019 | A |  | KR017814 | Human | Zhejiang, China | 2012 | A |
| LC570801 | Dog | Japan | 2019 | A |  | KU507550 | Human | South Korea | 2013 | A |  | KJ597823 | Human | Zhejiang, China | 2011 | A |
| AB817983 | Human | Japan | 2012 | A |  | OM160957 | Tick | South Korea | 2019 | A |  | KR017826 | Human | Zhejiang, China | 2014 | A |
| LC462235 | Cat | Japan | 2018 | A |  | OM160984 | Tick | South Korea | 2019 | A |  | MT236317 | Tick | Zhejiang, China | 2014 | A |
| AB983515 | Human | Japan | 2013 | B |  | KU507551 | Human | South Korea | 2013 | A |  | KU507553 | Human | South Korea | 2013 | A |
| JQ670934 | Human | Anhui, China | 2011 | B |  | AB985298 | Human | Japan | 2013 | A |  | AB985559 | Human | Japan | 2013 | A |
| AB983534 | Human | Japan | 2014 | B |  | AB985293 | Human | Japan | 2013 | A |  | AB817997 | Human | Japan | 2013 | A |
| LC579717 | Raccoon | Japan | 2013 | B |  | AB985301 | Human | Japan | 2013 | A |  | KC292292 | Human | Henan, China | 2012 | D |
| LC579720 | Raccoon | Japan | 2013 | B |  | MN995268 | Cat | Japan | 2019 | A |  | KC292293 | Human | Henan, China | 2012 | D |
| LC570798 | Dog | Japan | 2019 | B |  | AB985311 | Human | Japan | 2013 | A |  | KR612078 | Human | South Korea | 2013 | A |
| MH049431 | Tick | Jilin, China | 2017 | C |  | MZ773034 | Human | Zhejiang, China | 2020 | A |  | KR612076 | Human | South Korea | 2013 | A |
| KR230761 | Human | Jiangsu, China | 2014 | C |  | AB985299 | Human | Japan | 2013 | A |  | KU507576 | Human | South Korea | 2013 | A |
| KR017843 | Human | Zhejiang, China | 2013 | C |  | MN995271 | Cat | Japan | 2019 | A |  | KR612079 | Human | South Korea | 2013 | A |
| KR017839 | Human | Zhejiang, China | 2012 | C |  | OM179921 | Dog | South Korea | 2020 | A |  | KR612088 | Human | South Korea | 2013 | A |
| MZ773017 | Human | Zhejiang, China | 2020 | C |  | AB985322 | Human | Japan | 2012 | A |  | KR612087 | Human | South Korea | 2014 | A |
| MZ773016 | Human | Zhejiang, China | 2020 | C |  | LC570791 | Dog | Japan | 2019 | A |  | KR612083 | Human | South Korea | 2013 | A |
| MZ773025 | Human | Zhejiang, China | 2020 | C |  | AB985304 | Human | Japan | 2013 | A |  | KR612082 | Human | South Korea | 2014 | A |
| MZ773015 | Human | Zhejiang, China | 2020 | C |  | AB985307 | Human | Japan | 2013 | A |  | KR612084 | Human | South Korea | 2014 | A |
| MT114212 | Human | Liaoning, China | 2013 | C |  | KU507562 | Human | South Korea | 2013 | A |  | KR612086 | Human | South Korea | 2013 | A |
| HQ141610 | Human | Liaoning, China | 2010 | C |  | LC570788 | Dog | Japan | 2018 | A |  | KR612077 | Human | South Korea | 2014 | A |
| MT114203 | Human | Liaoning, China | 2011 | C |  | KU507560 | Human | South Korea | 2013 | A |  | KR612075 | Human | South Korea | 2014 | A |
| KT890280 | Tick | Jilin, China | 2013 | C |  | KP663741 | Human | South Korea | 2014 | A |  | MH937372 | Human | South Korea | 2017 | A |
| KY362316 | Human | Jiangsu, China | 2015 | C |  | KU507563 | Human | South Korea | 2013 | A |  | KR612085 | Human | South Korea | 2014 | A |
| KC505144 | Human | Jiangsu, China | 2012 | C |  | KP663735 | Human | South Korea | 2014 | A |  | KU507556 | Human | South Korea | 2013 | A |
| KC505138 | Human | Jiangsu, China | 2012 | C |  | AB985321 | Human | Japan | 2013 | A |  | KU507555 | Human | South Korea | 2013 | A |
| HQ141601 | Human | Jiangsu, China | 2010 | C |  | MN995266 | Cat | Japan | 2019 | A |  | KC292294 | Human | Henan, China | 2012 | F |
| KC505123 | Human | Jiangsu, China | 2012 | C |  | AB817990 | Human | Japan | 2012 | A |  | KC292296 | Human | Henan, China | 2012 | C |
| KR230759 | Human | Jiangsu, China | 2014 | C |  | AB817992 | Human | Japan | 2010 | A |  | AB985575 | Human | Japan | 2013 | A |
| MT005202 | Human | Shandong, China | 2011 | C |  | MN995280 | Cat | Japan | 2019 | A |  | AB985568 | Human | Japan | 2013 | A |
| KY362297 | Human | Jiangsu, China | 2011 | C |  | KU507561 | Human | South Korea | 2013 | A |  | AB985524 | Human | Japan | 2013 | A |
| KY362295 | Human | Jiangsu, China | 2011 | C |  | AB985302 | Human | Japan | 2013 | A |  | AB985534 | Human | Japan | 2013 | A |
| KY362296 | Human | Jiangsu, China | 2011 | C |  | KP663738 | Human | South Korea | 2014 | A |  | AB985529 | Human | Japan | 2013 | A |
| KY362294 | Human | Jiangsu, China | 2011 | C |  | AB817987 | Human | Japan | 2012 | A |  | MT114286 | Human | Hubei, China | 2012 | A |
| HQ141592 | Human | Anhui, China | 2010 | C |  | KU507567 | Human | South Korea | 2013 | A |  | AB985560 | Human | Japan | 2013 | A |
| MT005217 | Human | Shandong, China | 2012 | C |  | AB985308 | Human | Japan | 2013 | A |  | KC292298 | Human | Henan, China | 2012 | D |
| KC505129 | Human | Jiangsu, China | 2012 | C |  | AB985323 | Human | Japan | 2013 | A |  | AB985551 | Human | Japan | 2013 | A |
| KY362302 | Human | Jiangsu, China | 2012 | C |  | MN995276 | Cat | Japan | 2019 | A |  | AB985528 | Human | Japan | 2013 | A |
| KR230754 | Human | Jiangsu, China | 2013 | C |  | MN995277 | Cat | Japan | 2019 | A |  | KU507577 | Human | South Korea | 2013 | A |
| MT320799 | Human | Hubei, China | 2017 | C |  | MN995260 | Cat | Japan | 2019 | A |  | MN995282 | Cat | Japan | 2019 | A |
| OM453283 | Human | Henan, China | 2018 | C |  | AB817994 | Human | Japan | 2012 | A |  | MN995283 | Cat | Japan | 2019 | A |
| OM453080 | Human | Henan, China | 2014 | C |  | AB985317 | Human | Japan | 2013 | A |  | MN995284 | Cat | Japan | 2019 | A |
| OM453441 | Human | Henan, China | 2019 | C |  | LC536546 | Tick | Japan | 2016 | A |  | MN995285 | Cat | Japan | 2019 | A |
| MT114243 | Human | Hubei, China | 2013 | C |  | LC579713 | Raccoon | Japan | 2014 | A |  | AB985536 | Human | Japan | 2013 | A |
| OM453129 | Human | Henan, China | 2014 | C |  | LC462232 | Cat | Japan | 2018 | A |  | AB985548 | Human | Japan | 2013 | A |
| OM453491 | Human | Henan, China | 2020 | C |  | LC715250 | Cat | Japan | 2022 | A |  | AB985553 | Human | Japan | 2013 | A |
| MT114236 | Human | Hubei, China | 2012 | C |  | MN995269 | Cat | Japan | 2019 | A |  | MN995286 | Cat | Japan | 2019 | A |
| OM453057 | Human | Henan, China | 2013 | C |  | MN995259 | Cat | Japan | 2019 | A |  | LC570787 | Dog | Japan | 2018 | A |
| OM453252 | Human | Henan, China | 2016 | C |  | LC570800 | Dog | Japan | 2019 | A |  | KU507570 | Human | South Korea | 2013 | A |
| OM453471 | Human | Henan, China | 2020 | C |  | LC705155 | Cat | Japan | 2022 | A |  | AB985577 | Human | Japan | 2013 | A |
| OM453254 | Human | Henan, China | 2016 | C |  | AB985325 | Human | Japan | 2014 | A |  | KP663742 | Human | South Korea | 2014 | A |
| OM453362 | Human | Henan, China | 2018 | C |  | MN995254 | Cat | Japan | 2019 | A |  | KR612073 | Human | South Korea | 2013 | A |
| OM453436 | Human | Henan, China | 2019 | C |  | MN995281 | Cat | Japan | 2019 | A |  | KR612074 | Human | South Korea | 2013 | A |
| OM453047 | Human | Henan, China | 2013 | C |  | MN995272 | Cat | Japan | 2019 | A |  | KR612072 | Human | South Korea | 2013 | A |
| MN509950 | Human | Henan, China | 2015 | C |  | MN995263 | Cat | Japan | 2019 | A |  | KU507572 | Human | South Korea | 2013 | A |
| OM453074 | Human | Henan, China | 2014 | C |  | MN995278 | Cat | Japan | 2019 | A |  | KP663736 | Human | South Korea | 2014 | A |
| OM453258 | Human | Henan, China | 2016 | C |  | MN995255 | Cat | Japan | 2019 | A |  | KU507573 | Human | South Korea | 2013 | A |
| OM453060 | Human | Henan, China | 2013 | C |  | MN995253 | Cat | Japan | 2019 | A |  | AB985557 | Human | Japan | 2013 | A |
| OM453101 | Human | Henan, China | 2014 | C |  | MN995262 | Cat | Japan | 2019 | A |  | AB817998 | Human | Japan | 2013 | A |
| KF791960 | Tick | Henan, China | 2013 | C |  | MN995256 | Cat | Japan | 2019 | A |  | AB818001 | Human | Japan | 2013 | A |
| OM453347 | Human | Henan, China | 2018 | C |  | MN995279 | Cat | Japan | 2019 | A |  | MN995287 | Cat | Japan | 2019 | A |
| OM453458 | Human | Henan, China | 2020 | C |  | MN995261 | Cat | Japan | 2019 | A |  | KU507571 | Human | South Korea | 2013 | A |
| OM453398 | Human | Henan, China | 2017 | C |  | JQ670930 | Human | Anhui, China | 2011 | B |  | AB985533 | Human | Japan | 2013 | A |
| OM453507 | Human | Henan, China | 2020 | C |  | MT114256 | Human | Hubei, China | 2012 | B |  | AB817995 | Human | Japan | 2013 | A |
| OM453607 | Human | Henan, China | 2019 | C |  | LC579716 | Raccoon | Japan | 2013 | B |  | MN995288 | Cat | Japan | 2019 | A |
| OM453542 | Human | Henan, China | 2020 | C |  | LC590891 | Human | Japan | 2017 | B |  | AB817996 | Human | Japan | 2013 | A |
| OM453372 | Human | Henan, China | 2017 | C |  | LC579719 | Raccoon | Japan | 2013 | B |  | LC462229 | Cat | Japan | 2018 | A |
| OM453255 | Human | Henan, China | 2016 | C |  | LC570797 | Dog | Japan | 2019 | B |  | MN995289 | Cat | Japan | 2019 | A |
| HQ171190 | Human | Hubei, China | 2010 | C |  | KR230781 | Human | Jiangsu, China | 2014 | C |  | MN995290 | Cat | Japan | 2019 | A |
| OM453360 | Human | Henan, China | 2018 | C |  | KU507564 | Human | South Korea | 2013 | C |  | MN995291 | Cat | Japan | 2019 | A |
| OM453448 | Human | Henan, China | 2020 | C |  | KR017858 | Human | Zhejiang, China | 2012 | C |  | MN995292 | Cat | Japan | 2019 | A |
| OM453501 | Human | Henan, China | 2020 | C |  | KR017862 | Human | Zhejiang, China | 2013 | C |  | AB985573 | Human | Japan | 2013 | A |
| OM453109 | Human | Henan, China | 2014 | C |  | MZ773040 | Human | Zhejiang, China | 2020 | C |  | MN995293 | Cat | Japan | 2019 | A |
| OM453043 | Human | Henan, China | 2013 | C |  | MZ773030 | Human | Zhejiang, China | 2020 | C |  | MN995294 | Cat | Japan | 2019 | A |
| OM453204 | Human | Henan, China | 2015 | C |  | KU507565 | Human | South Korea | 2013 | C |  | MN995295 | Cat | Japan | 2019 | A |
| OM453449 | Human | Jiangxi, China | 2020 | C |  | LC570785 | Dog | Japan | 2018 | C |  | MN995296 | Cat | Japan | 2019 | A |
| MT114239 | Human | Hubei, China | 2012 | C |  | KR698336 | Human | Zhejiang, China | 2013 | C |  | MN995297 | Cat | Japan | 2019 | A |
| MT320796 | Human | Hubei, China | 2017 | C |  | KR698341 | Human | Zhejiang, China | 2013 | C |  | MN995298 | Cat | Japan | 2019 | A |
| MT005223 | Human | Shandong, China | 2012 | C |  | MZ773031 | Human | Zhejiang, China | 2020 | C |  | MN995299 | Cat | Japan | 2019 | A |
| OM452944 | Human | Henan, China | 2012 | C |  | MZ773032 | Human | Zhejiang, China | 2020 | C |  | MN995300 | Cat | Japan | 2019 | A |
| OM453071 | Human | Henan, China | 2014 | C |  | MZ561691 | Human | Zhejiang, China | 2018 | C |  | MN995301 | Cat | Japan | 2019 | A |
| OM453002 | Human | Henan, China | 2013 | C |  | MT114220 | Human | Liaoning, China | 2011 | C |  | MN995302 | Cat | Japan | 2019 | A |
| OM453399 | Human | Henan, China | 2018 | C |  | MT114228 | Human | Liaoning, China | 2013 | C |  | MN995304 | Cat | Japan | 2019 | A |
| OM453447 | Human | Henan, China | 2020 | C |  | KY362364 | Human | Jiangsu, China | 2015 | C |  | MN995306 | Cat | Japan | 2019 | A |
| OM453119 | Human | Henan, China | 2014 | C |  | KR230779 | Human | Jiangsu, China | 2014 | C |  | AB985541 | Human | Japan | 2013 | B |
| OM453610 | Human | Henan, China | 2019 | C |  | KC505145 | Human | Jiangsu, China | 2012 | C |  | LC579715 | Raccoon | Japan | 2013 | B |
| OM453041 | Human | Henan, China | 2013 | C |  | KR230777 | Human | Jiangsu, China | 2013 | C |  | AB985572 | Human | Japan | 2013 | B |
| OM453020 | Human | Henan, China | 2013 | C |  | MT005244 | Human | Shandong, China | 2011 | C |  | LC579718 | Raccoon | Japan | 2013 | B |
| OM453544 | Human | Henan, China | 2019 | C |  | JF837594 | Human | Jiangsu, China | 2011 | C |  | KR230801 | Human | Jiangsu, China | 2014 | C |
| OM453170 | Human | Henan, China | 2015 | C |  | KR230770 | Human | Jiangsu, China | 2011 | C |  | MH177014 | Tick | Jilin, China | 2017 | C |
| OM453121 | Human | Henan, China | 2014 | C |  | KC505142 | Human | Jiangsu, China | 2012 | C |  | KT890282 | Tick | Jilin, China | 2013 | C |
| OM453290 | Human | Henan, China | 2018 | C |  | KC505124 | Human | Jiangsu, China | 2012 | C |  | KR017824 | Human | Zhejiang, China | 2013 | C |
| KY965118 | Human | Hubei, China | 2016 | C |  | KY362342 | Human | Jiangsu, China | 2011 | C |  | KR017816 | Human | Zhejiang, China | 2012 | C |
| MT114240 | Human | Hubei, China | 2012 | C |  | KY362345 | Human | Jiangsu, China | 2011 | C |  | MZ773046 | Human | Zhejiang, China | 2020 | C |
| OM453298 | Human | Henan, China | 2017 | C |  | KY362344 | Human | Jiangsu, China | 2011 | C |  | KU507575 | Human | South Korea | 2013 | C |
| OM453151 | Human | Henan, China | 2014 | C |  | KR230774 | Human | Jiangsu, China | 2013 | C |  | LC570784 | Dog | Japan | 2018 | C |
| OM453085 | Human | Henan, China | 2014 | C |  | KY362343 | Human | Jiangsu, China | 2011 | C |  | MZ773057 | Human | Zhejiang, China | 2020 | C |
| OM453295 | Human | Henan, China | 2018 | C |  | KC505130 | Human | Jiangsu, China | 2012 | C |  | MZ773055 | Human | Zhejiang, China | 2020 | C |
| OM453262 | Human | Henan, China | 2016 | C |  | KY362347 | Human | Jiangsu, China | 2011 | C |  | MZ773047 | Human | Zhejiang, China | 2020 | C |
| OM452998 | Human | Henan, China | 2013 | C |  | MT005262 | Human | Shandong, China | 2013 | C |  | KR698323 | Human | Zhejiang, China | 2013 | C |
| OM452969 | Human | Henan, China | 2013 | C |  | MT005261 | Human | Shandong, China | 2013 | C |  | MZ773045 | Human | Zhejiang, China | 2020 | C |
| OM453142 | Human | Henan, China | 2014 | C |  | OM452532 | Human | Henan, China | 2015 | C |  | MZ561690 | Human | Zhejiang, China | 2022 | C |
| MN509929 | Human | Henan, China | 2015 | C |  | OM452630 | Human | Henan, China | 2018 | C |  | MT114272 | Human | Liaoning, China | 2013 | C |
| OM453401 | Human | Henan, China | 2017 | C |  | OM452395 | Human | Henan, China | 2014 | C |  | MT114264 | Human | Liaoning, China | 2011 | C |
| OM453468 | Human | Henan, China | 2020 | C |  | OM452337 | Human | Henan, China | 2013 | C |  | KR230799 | Human | Jiangsu, China | 2014 | C |
| OM453311 | Human | Henan, China | 2018 | C |  | OM452261 | Human | Henan, China | 2012 | C |  | KR230797 | Human | Jiangsu, China | 2013 | C |
| MT005200 | Human | Shandong, China | 2011 | D |  | OM452349 | Human | Henan, China | 2013 | C |  | MT114292 | Human | Shandong, China | 2011 | C |
| MT005218 | Human | Shandong, China | 2013 | D |  | OM452595 | Human | Henan, China | 2017 | C |  | KY362340 | Human | Jiangsu, China | 2015 | C |
| MT005209 | Human | Shandong, China | 2011 | D |  | OM452283 | Human | Henan, China | 2013 | C |  | KC505125 | Human | Jiangsu, China | 2012 | C |
| KY965120 | Human | Hubei, China | 2016 | D |  | OM452664 | Human | Henan, China | 2018 | C |  | KC505146 | Human | Jiangsu, China | 2012 | C |
| KY965122 | Human | Hubei, China | 2016 | D |  | OM452717 | Human | Henan, China | 2017 | C |  | HQ141603 | Human | Jiangsu, China | 2010 | C |
| KY965117 | Human | Hubei, China | 2016 | D |  | OM452583 | Human | Henan, China | 2016 | C |  | KR230790 | Human | Jiangsu, China | 2011 | C |
| MT005213 | Human | Shandong, China | 2012 | D |  | KF711928 | Human | Henan, China | 2011 | C |  | KY362329 | Human | Jiangsu, China | 2014 | C |
| KY362303 | Human | Jiangsu, China | 2013 | D |  | OM452672 | Human | Henan, China | 2017 | C |  | KY362320 | Human | Jiangsu, China | 2011 | C |
| MT005230 | Human | Shandong, China | 2014 | D |  | OM452928 | Human | Henan, China | 2019 | C |  | HQ830171 | Human | Jiangsu, China | 2010 | C |
| MT114210 | Human | Liaoning, China | 2013 | D |  | OM452916 | Human | Henan, China | 2019 | C |  | MT114310 | Human | Shandong, China | 2013 | C |
| MT114200 | Human | Liaoning, China | 2010 | D |  | OM452246 | Human | Henan, China | 2011 | C |  | MT114309 | Human | Shandong, China | 2013 | C |
| MT114214 | Human | Liaoning, China | 2013 | D |  | MT320800 | Human | Hubei, China | 2017 | C |  | MT114307 | Human | Shandong, China | 2012 | C |
| KF358691 | Human | South Korea | 2012 | D |  | KF791953 | Human | Henan, China | 2013 | C |  | KC505131 | Human | Jiangsu, China | 2012 | C |
| KR017830 | Human | Anhui, China | 2012 | D |  | OM452408 | Human | Henan, China | 2014 | C |  | KR230794 | Human | Jiangsu, China | 2013 | C |
| KY965115 | Human | Hubei, China | 2016 | D |  | OM452255 | Human | Henan, China | 2012 | C |  | KR230804 | Human | Jiangsu, China | 2014 | C |
| HQ171189 | Human | Hubei, China | 2010 | D |  | KF711929 | Human | Henan, China | 2012 | C |  | KU664014 | Human | Hubei, China | 2012 | C |
| OM453546 | Human | Henan, China | 2019 | D |  | OM452579 | Human | Henan, China | 2016 | C |  | HQ141594 | Human | Anhui, China | 2010 | C |
| KC292333 | Human | Henan, China | 2011 | D |  | OM452509 | Human | Henan, China | 2015 | C |  | KY362323 | Human | Jiangsu, China | 2011 | C |
| MN509905 | Human | Henan, China | 2014 | D |  | HQ419236 | Human | Hubei, China | 2010 | C |  | KY362318 | Human | Jiangsu, China | 2011 | C |
| OM453565 | Human | Henan, China | 2019 | D |  | OM452651 | Human | Henan, China | 2018 | C |  | MN510215 | Human | Henan, China | 2013 | C |
| OM453439 | Human | Henan, China | 2019 | D |  | OM452582 | Human | Henan, China | 2016 | C |  | MN510211 | Human | Henan, China | 2013 | C |
| MN509972 | Human | Henan, China | 2016 | D |  | OM452320 | Human | Henan, China | 2013 | C |  | MN510228 | Human | Henan, China | 2014 | C |
| KF356552 | Human | Henan, China | 2011 | D |  | MT114254 | Human | Hubei, China | 2012 | C |  | MT320798 | Human | Hubei, China | 2017 | C |
| OM453281 | Human | Hubei, China | 2017 | D |  | OM452493 | Human | Henan, China | 2015 | C |  | KP339884 | Human | Henan, China | 2014 | C |
| OM453269 | Human | Henan, China | 2016 | D |  | OM452436 | Human | Henan, China | 2014 | C |  | OM451917 | Human | Henan, China | 2018 | C |
| MN509976 | Human | Henan, China | 2017 | D |  | OM452611 | Human | Henan, China | 2018 | C |  | KR075912 | Human | Henan, China | 2014 | C |
| OM453590 | Human | Henan, China | 2019 | D |  | MT320797 | Human | Hubei, China | 2017 | C |  | OM451745 | Human | Henan, China | 2014 | C |
| MN509964 | Human | Henan, China | 2016 | D |  | OM452313 | Human | Henan, China | 2013 | C |  | MT114283 | Human | Hubei, China | 2012 | C |
| OM453240 | Human | Henan, China | 2016 | D |  | OM452618 | Human | Henan, China | 2017 | C |  | OM452109 | Human | Henan, China | 2020 | C |
| MN509845 | Human | Henan, China | 2011 | D |  | OM452586 | Human | Henan, China | 2016 | C |  | OM451673 | Human | Henan, China | 2013 | C |
| KY965114 | Human | Hubei, China | 2016 | D |  | OM452399 | Human | Henan, China | 2014 | C |  | KP339903 | Human | Henan, China | 2014 | C |
| KF711870 | Human | Henan, China | 2011 | D |  | OM452462 | Human | Henan, China | 2014 | C |  | MT114313 | Human | Shandong, China | 2013 | C |
| MN509875 | Human | Hubei, China | 2013 | D |  | OM452317 | Human | Henan, China | 2013 | C |  | MN510221 | Human | Henan, China | 2013 | C |
| OM453484 | Human | Henan, China | 2020 | D |  | OM452300 | Human | Henan, China | 2013 | C |  | OM452099 | Human | Henan, China | 2020 | C |
| OM453464 | Human | Henan, China | 2020 | D |  | KC292301 | Human | Henan, China | 2012 | C |  | MT114284 | Human | Hubei, China | 2012 | C |
| OM453495 | Human | Henan, China | 2020 | D |  | OM452540 | Human | Henan, China | 2015 | C |  | OM452166 | Human | Henan, China | 2019 | C |
| KF356545 | Human | Henan, China | 2011 | D |  | OM452527 | Human | Henan, China | 2015 | C |  | KT721303 | Human | Henan, China | 2015 | C |
| KF356543 | Human | Henan, China | 2011 | D |  | OM452248 | Human | Henan, China | 2012 | C |  | OM451682 | Human | Henan, China | 2013 | C |
| MN509911 | Human | Henan, China | 2014 | D |  | MT005265 | Human | Shandong, China | 2013 | C |  | MN510234 | Human | Hubei, China | 2014 | C |
| OM453250 | Human | Henan, China | 2016 | D |  | OM452808 | Human | Henan, China | 2020 | C |  | OM451648 | Human | Henan, China | 2013 | C |
| MN509937 | Human | Henan, China | 2015 | D |  | OM452584 | Human | Henan, China | 2016 | C |  | OM452023 | Human | Henan, China | 2018 | C |
| OM452967 | Human | Henan, China | 2013 | D |  | MT114253 | Human | Hubei, China | 2012 | C |  | OM452069 | Human | Henan, China | 2020 | C |
| MN509893 | Human | Hubei, China | 2013 | D |  | OM452303 | Human | Henan, China | 2013 | C |  | KF356523 | Human | Henan, China | 2011 | C |
| OM453606 | Human | Hubei, China | 2019 | D |  | OM452247 | Human | Henan, China | 2012 | C |  | MN510225 | Human | Henan, China | 2013 | C |
| OM453349 | Human | Henan, China | 2017 | D |  | OM452409 | Human | Henan, China | 2014 | C |  | MN510230 | Human | Henan, China | 2014 | C |
| OM453337 | Human | Henan, China | 2018 | D |  | OM452768 | Human | Henan, China | 2020 | C |  | MN510218 | Human | Henan, China | 2013 | C |
| OM453527 | Human | Hubei, China | 2020 | D |  | OM452931 | Human | Henan, China | 2019 | C |  | KP339911 | Human | Henan, China | 2014 | C |
| OM453264 | Human | Henan, China | 2016 | D |  | KF356534 | Human | Henan, China | 2011 | C |  | OM451711 | Human | Henan, China | 2014 | C |
| MN510010 | Human | Henan, China | 2018 | D |  | OM452568 | Human | Henan, China | 2016 | C |  | OM451619 | Human | Henan, China | 2013 | C |
| OM453555 | Human | Henan, China | 2019 | D |  | OM452718 | Human | Henan, China | 2018 | C |  | KP339912 | Human | Henan, China | 2014 | C |
| MN509851 | Human | Henan, China | 2012 | D |  | OM452404 | Human | Henan, China | 2014 | C |  | OM452025 | Human | Henan, China | 2017 | C |
| OM453328 | Human | Henan, China | 2018 | D |  | OM452323 | Human | Henan, China | 2013 | C |  | MN510220 | Human | Henan, China | 2013 | C |
| OM453469 | Human | Hubei, China | 2020 | D |  | OM452400 | Human | Henan, China | 2014 | C |  | OM451922 | Human | Henan, China | 2018 | C |
| OM453395 | Human | Hubei, China | 2018 | D |  | KC292323 | Human | Henan, China | 2012 | C |  | OM451694 | Human | Henan, China | 2014 | C |
| OM453455 | Human | Hubei, China | 2020 | D |  | OM452559 | Human | Henan, China | 2016 | C |  | OM451737 | Human | Henan, China | 2014 | C |
| OM453045 | Human | Henan, China | 2013 | D |  | OM452604 | Human | Henan, China | 2018 | C |  | OM451697 | Human | Henan, China | 2014 | C |
| OM453444 | Human | Hubei, China | 2019 | D |  | OM452386 | Human | Henan, China | 2014 | C |  | OM451810 | Human | Henan, China | 2015 | C |
| MN509910 | Human | Hubei, China | 2014 | D |  | OM452657 | Human | Henan, China | 2018 | C |  | OM451759 | Human | Henan, China | 2014 | C |
| KF356544 | Human | Henan, China | 2011 | D |  | OM452822 | Human | Henan, China | 2020 | C |  | OM451805 | Human | Henan, China | 2015 | C |
| OM453356 | Human | Henan, China | 2017 | D |  | OM452863 | Human | Henan, China | 2020 | C |  | OM451712 | Human | Henan, China | 2014 | C |
| MN509865 | Human | Hubei, China | 2012 | D |  | OM452684 | Human | Henan, China | 2018 | C |  | OM451704 | Human | Henan, China | 2014 | C |
| MN509985 | Human | Hubei, China | 2017 | D |  | MT114250 | Human | Hubei, China | 2012 | C |  | OM451731 | Human | Henan, China | 2014 | C |
| OM452979 | Human | Henan, China | 2013 | D |  | OM452796 | Human | Henan, China | 2020 | C |  | MN510247 | Human | Henan, China | 2015 | C |
| KC292334 | Human | Henan, China | 2011 | D |  | OM452576 | Human | Henan, China | 2016 | C |  | OM451738 | Human | Henan, China | 2014 | C |
| OM453344 | Human | Henan, China | 2018 | D |  | OM452363 | Human | Henan, China | 2013 | C |  | OM451708 | Human | Henan, China | 2014 | C |
| OM453460 | Human | Henan, China | 2020 | D |  | OM452249 | Human | Hubei, China | 2012 | C |  | OM451817 | Human | Henan, China | 2015 | C |
| OM453499 | Human | Henan, China | 2020 | D |  | MT114257 | Human | Hubei, China | 2013 | C |  | OM451799 | Human | Henan, China | 2015 | C |
| OM453385 | Human | Henan, China | 2018 | D |  | OM452812 | Human | Henan, China | 2020 | C |  | OM451957 | Human | Henan, China | 2017 | C |
| OM453608 | Human | Hubei, China | 2019 | D |  | OM452318 | Human | Henan, China | 2013 | C |  | MN510187 | Human | Henan, China | 2012 | C |
| OM453292 | Human | Hubei, China | 2017 | D |  | OM452757 | Human | Henan, China | 2019 | C |  | MN510204 | Human | Henan, China | 2012 | C |
| OM453559 | Human | Hubei, China | 2019 | D |  | OM452803 | Human | Henan, China | 2020 | C |  | KF917446 | Human | Henan, China | 2013 | C |
| OM453554 | Human | Hubei, China | 2019 | D |  | OM452554 | Human | Henan, China | 2016 | C |  | OM451865 | Human | Henan, China | 2015 | C |
| KC292332 | Human | Henan, China | 2011 | D |  | OM452857 | Human | Henan, China | 2020 | C |  | OM451726 | Human | Henan, China | 2014 | C |
| OM453379 | Human | Henan, China | 2017 | D |  | KC292322 | Human | Henan, China | 2012 | C |  | HQ419244 | Human | Henan, China | 2010 | C |
| MN509860 | Human | Henan, China | 2012 | D |  | OM452521 | Human | Henan, China | 2015 | C |  | OM451696 | Human | Henan, China | 2014 | C |
| OM453302 | Human | Henan, China | 2018 | D |  | OM452600 | Human | Henan, China | 2017 | C |  | MT114280 | Human | Hubei, China | 2012 | C |
| OM453586 | Human | Hubei, China | 2019 | D |  | OM452326 | Human | Henan, China | 2013 | C |  | OM452164 | Human | Henan, China | 2020 | C |
| MN509855 | Human | Henan, China | 2012 | D |  | OM452340 | Human | Henan, China | 2013 | C |  | MN995308 | Cat | Japan | 2019 | A |
| OM453148 | Human | Henan, China | 2014 | D |  | OM452578 | Human | Henan, China | 2016 | C |  | MN510317 | Human | Henan, China | 2018 | C |
| OM453408 | Human | Henan, China | 2018 | D |  | OM452688 | Human | Henan, China | 2017 | C |  | KP339924 | Human | Henan, China | 2014 | C |
| OM453386 | Human | Henan, China | 2017 | D |  | OM452353 | Human | Henan, China | 2013 | C |  | MN510303 | Human | Henan, China | 2018 | C |
| KY440774 | Human | Hubei, China | 2015 | D |  | OM452891 | Human | Henan, China | 2019 | C |  | KP339935 | Human | Henan, China | 2014 | C |
| MN509900 | Human | Hubei, China | 2014 | D |  | OM452878 | Human | Henan, China | 2019 | C |  | KF917445 | Human | Henan, China | 2013 | C |
| MN509877 | Human | Hubei, China | 2013 | D |  | OM452581 | Human | Henan, China | 2016 | C |  | KP339914 | Human | Henan, China | 2014 | C |
| OM453267 | Human | Henan, China | 2016 | D |  | OM452817 | Human | Henan, China | 2020 | C |  | OM452192 | Human | Henan, China | 2019 | C |
| OM453601 | Human | Hubei, China | 2019 | D |  | OM452834 | Human | Henan, China | 2020 | C |  | KP339892 | Human | Henan, China | 2014 | C |
| OM453509 | Human | Hubei, China | 2020 | D |  | OM452631 | Human | Henan, China | 2018 | C |  | OM452158 | Human | Henan, China | 2020 | C |
| OM453609 | Human | Hubei, China | 2019 | D |  | OM452892 | Human | Henan, China | 2019 | C |  | OM452024 | Human | Henan, China | 2018 | C |
| MN509890 | Human | Henan, China | 2013 | D |  | OM452824 | Human | Henan, China | 2020 | C |  | OM452093 | Human | Henan, China | 2020 | C |
| OM453094 | Human | Henan, China | 2014 | D |  | OM452680 | Human | Henan, China | 2018 | C |  | OM451867 | Human | Henan, China | 2016 | C |
| OM453461 | Human | Henan, China | 2020 | D |  | OM452571 | Human | Henan, China | 2016 | C |  | KT380647 | Human | Henan, China | 2015 | C |
| MT114245 | Human | Hubei, China | 2014 | D |  | MT005268 | Human | Shandong, China | 2013 | D |  | OM452104 | Human | Henan, China | 2020 | C |
| MN509881 | Human | Henan, China | 2013 | D |  | KY965102 | Human | Hubei, China | 2016 | D |  | KT721302 | Human | Henan, China | 2015 | C |
| OM453164 | Human | Henan, China | 2015 | D |  | KY965104 | Human | Hubei, China | 2016 | D |  | OM452125 | Human | Henan, China | 2020 | C |
| OM453287 | Human | Henan, China | 2017 | D |  | KY965099 | Human | Hubei, China | 2016 | D |  | OM452172 | Human | Henan, China | 2019 | C |
| OM453376 | Human | Henan, China | 2017 | D |  | MT005255 | Human | Shandong, China | 2012 | D |  | OM451889 | Human | Henan, China | 2016 | C |
| OM453313 | Human | Henan, China | 2018 | D |  | KY362351 | Human | Jiangsu, China | 2013 | D |  | HQ419241 | Human | Henan, China | 2010 | C |
| OM453584 | Human | Henan, China | 2019 | D |  | MT005272 | Human | Shandong, China | 2014 | D |  | KU738910 | Human | Hubei, China | 2014 | C |
| OM453576 | Human | Henan, China | 2019 | D |  | MT005242 | Human | Shandong, China | 2011 | D |  | MN510289 | Human | Henan, China | 2017 | C |
| KF356550 | Human | Henan, China | 2011 | D |  | MT005275 | Human | Shandong, China | 2014 | D |  | MN510212 | Human | Henan, China | 2013 | C |
| OM453319 | Human | Hubei, China | 2018 | D |  | MT005260 | Human | Shandong, China | 2013 | D |  | OM451666 | Human | Henan, China | 2013 | C |
| OM453504 | Human | Hubei, China | 2020 | D |  | MT005251 | Human | Shandong, China | 2011 | D |  | HQ171195 | Human | Hubei, China | 2010 | C |
| OM452978 | Human | Henan, China | 2013 | D |  | HM802203 | Human | Shandong, China | 2010 | D |  | OM451681 | Human | Henan, China | 2013 | C |
| OM453336 | Human | Henan, China | 2018 | D |  | MN510115 | Human | Henan, China | 2016 | D |  | MN510319 | Human | Henan, China | 2018 | C |
| OM453572 | Human | Henan, China | 2019 | D |  | KF887436 | Human | Liaoning, China | 2012 | D |  | OM451566 | Human | Hubei, China | 2012 | C |
| MW021168 | Human | Henan, China | 2020 | D |  | KX302599 | Human | Anhui, China | 2015 | D |  | MT114287 | Human | Hubei, China | 2013 | C |
| OM453261 | Human | Hubei, China | 2016 | D |  | MT114224 | Human | Liaoning, China | 2012 | D |  | KT736098 | Human | Henan, China | 2015 | C |
| OM453359 | Human | Henan, China | 2018 | D |  | KF887439 | Human | Liaoning, China | 2012 | D |  | MN510237 | Human | Henan, China | 2014 | C |
| OM453160 | Human | Henan, China | 2015 | D |  | MT114219 | Human | Liaoning, China | 2011 | D |  | OM452113 | Human | Henan, China | 2020 | C |
| OM453522 | Human | Henan, China | 2020 | D |  | MT114229 | Human | Liaoning, China | 2013 | D |  | OM451911 | Human | Henan, China | 2018 | C |
| OM453084 | Human | Henan, China | 2014 | D |  | MT114222 | Human | Liaoning, China | 2012 | D |  | OM452070 | Human | Henan, China | 2020 | C |
| JF682773 | Human | Henan, China | 2010 | D |  | MT114218 | Human | Liaoning, China | 2011 | D |  | KF711901 | Human | Henan, China | 2012 | C |
| HQ642766 | Human | Henan, China | 2010 | D |  | HQ141608 | Human | Liaoning, China | 2010 | D |  | HQ419240 | Human | Henan, China | 2010 | C |
| OM453450 | Human | Henan, China | 2020 | D |  | HQ419238 | Human | Hubei, China | 2010 | D |  | OM451877 | Human | Henan, China | 2016 | C |
| MN509904 | Human | Henan, China | 2014 | D |  | KF887440 | Human | Liaoning, China | 2012 | D |  | OM451901 | Human | Henan, China | 2016 | C |
| OM453227 | Human | Henan, China | 2015 | D |  | MT114227 | Human | Liaoning, China | 2013 | D |  | OM451792 | Human | Henan, China | 2015 | C |
| MT114209 | Human | Liaoning, China | 2012 | D |  | KY965098 | Human | Hubei, China | 2016 | D |  | OM451744 | Human | Henan, China | 2014 | C |
| JQ670929 | Human | Anhui, China | 2011 | D |  | MT114248 | Human | Hubei, China | 2011 | D |  | OM451913 | Human | Henan, China | 2018 | C |
| OM453556 | Human | Henan, China | 2019 | D |  | OM452858 | Human | Henan, China | 2020 | D |  | OM451776 | Human | Henan, China | 2014 | C |
| KR230763 | Human | Jiangsu, China | 2014 | D |  | OM452897 | Human | Henan, China | 2019 | D |  | OM451620 | Human | Henan, China | 2013 | C |
| MT114211 | Human | Liaoning, China | 2013 | D |  | OM452930 | Human | Hubei, China | 2019 | D |  | OM452022 | Human | Henan, China | 2017 | C |
| KY965116 | Human | Hubei, China | 2016 | D |  | MN510064 | Human | Henan, China | 2013 | D |  | OM452102 | Human | Henan, China | 2020 | C |
| MT114234 | Human | Hubei, China | 2011 | D |  | OM452883 | Human | Henan, China | 2019 | D |  | OM451972 | Human | Henan, China | 2017 | C |
| MT114206 | Human | Liaoning, China | 2012 | D |  | OM452866 | Human | Henan, China | 2019 | D |  | KP339919 | Human | Henan, China | 2014 | C |
| MT114202 | Human | Liaoning, China | 2011 | D |  | OM452633 | Human | Henan, China | 2018 | D |  | KP339936 | Human | Henan, China | 2014 | C |
| JF906056 | Tick | Hubei, China | 2010 | D |  | OM452932 | Human | Henan, China | 2019 | D |  | OM452129 | Human | Henan, China | 2020 | C |
| HQ141607 | Human | Liaoning, China | 2010 | D |  | OM452679 | Human | Henan, China | 2018 | D |  | OM452190 | Human | Henan, China | 2019 | C |
| JQ317178 | Human | Jiangsu, China | 2011 | D |  | OM452277 | Human | Henan, China | 2013 | D |  | MT114317 | Human | Shandong, China | 2013 | D |
| KR230766 | Tick | Jiangsu, China | 2014 | D |  | OM452693 | Human | Henan, China | 2017 | D |  | MT114290 | Human | Shandong, China | 2011 | D |
| KR230762 | Human | Jiangsu, China | 2014 | D |  | OM452495 | Human | Henan, China | 2015 | D |  | HM802204 | Human | Shandong, China | 2010 | D |
| KY362307 | Human | Jiangsu, China | 2015 | D |  | OM452501 | Human | Henan, China | 2015 | D |  | MT114299 | Human | Shandong, China | 2011 | D |
| KR230756 | Human | Jiangsu, China | 2013 | D |  | OM452401 | Human | Henan, China | 2014 | D |  | MT114323 | Human | Shandong, China | 2014 | D |
| KC505126 | Human | Jiangsu, China | 2012 | D |  | OM452268 | Human | Henan, China | 2013 | D |  | MT114308 | Human | Shandong, China | 2013 | D |
| KR230764 | Human | Jiangsu, China | 2014 | D |  | OM452665 | Human | Henan, China | 2018 | D |  | MT114303 | Human | Shandong, China | 2012 | D |
| JQ317169 | Human | Jiangsu, China | 2010 | D |  | OM452572 | Human | Henan, China | 2016 | D |  | KY362327 | Human | Jiangsu, China | 2013 | D |
| KY362304 | Human | Jiangsu, China | 2013 | D |  | OM452627 | Human | Henan, China | 2018 | D |  | MT114320 | Human | Shandong, China | 2014 | D |
| KC473537 | Goat | Jiangsu, China | 2012 | D |  | MN510040 | Human | Henan, China | 2012 | D |  | KU664017 | Human | Hubei, China | 2013 | D |
| KC473540 | Tick | Jiangsu, China | 2012 | D |  | OM452806 | Human | Henan, China | 2020 | D |  | KY965085 | Human | Hubei, China | 2016 | D |
| KR230769 | Hedgehog | Jiangsu, China | 2014 | D |  | OM452667 | Human | Henan, China | 2017 | D |  | KY965087 | Human | Hubei, China | 2016 | D |
| KR706567 | Human | Hubei, China | 2014 | D |  | KF356539 | Human | Henan, China | 2012 | D |  | KY965082 | Human | Hubei, China | 2016 | D |
| JQ684871 | Tick | Shandong, China | 2010 | D |  | KF711943 | Human | Henan, China | 2012 | D |  | OM452230 | Human | Hubei, China | 2019 | D |
| JQ317175 | Human | Jiangsu, China | 2010 | D |  | OM452699 | Human | Henan, China | 2018 | D |  | MT114270 | Human | Liaoning, China | 2013 | D |
| KR230758 | Human | Jiangsu, China | 2014 | D |  | OM452832 | Human | Henan, China | 2020 | D |  | MT114260 | Human | Liaoning, China | 2010 | D |
| KC505132 | Human | Jiangsu, China | 2012 | D |  | OM452711 | Human | Henan, China | 2018 | D |  | MT114274 | Human | Liaoning, China | 2013 | D |
| JF267783 | Dog | Jiangsu, China | 2011 | D |  | KC292303 | Human | Henan, China | 2012 | D |  | HQ171193 | Human | Hubei, China | 2010 | D |
| KR230755 | Human | Jiangsu, China | 2013 | D |  | OM452550 | Human | Henan, China | 2015 | D |  | HQ171194 | Human | Hubei, China | 2010 | D |
| KY362301 | Human | Jiangsu, China | 2011 | D |  | MN510111 | Human | Henan, China | 2016 | D |  | KR017811 | Human | Anhui, China | 2012 | D |
| KY362298 | Human | Jiangsu, China | 2011 | D |  | OM452771 | Human | Henan, China | 2020 | D |  | MT114277 | Human | Hubei, China | 2011 | D |
| KY362311 | Human | Jiangsu, China | 2013 | D |  | OM452862 | Human | Henan, China | 2020 | D |  | KF887435 | Human | Liaoning, China | 2012 | D |
| KY362309 | Human | Jiangsu, China | 2015 | D |  | OM452492 | Human | Henan, China | 2015 | D |  | MT114266 | Human | Liaoning, China | 2012 | D |
| KY362300 | Human | Jiangsu, China | 2011 | D |  | MW021169 | Human | Henan, China | 2020 | D |  | MT114262 | Human | Liaoning, China | 2011 | D |
| OM453582 | Human | Henan, China | 2019 | D |  | OM452305 | Human | Henan, China | 2013 | D |  | JF906058 | Tick | Hubei, China | 2010 | D |
| OM453353 | Human | Henan, China | 2018 | D |  | OM452733 | Human | Henan, China | 2017 | D |  | KU664008 | Human | Hubei, China | 2012 | D |
| MN509841 | Human | Henan, China | 2011 | D |  | OM452585 | Human | Hubei, China | 2016 | D |  | OM452212 | Human | Henan, China | 2019 | D |
| OM453066 | Human | Henan, China | 2014 | D |  | OM452575 | Human | Henan, China | 2016 | D |  | MN510288 | Human | Hubei, China | 2017 | D |
| OM453373 | Human | Henan, China | 2018 | D |  | OM452309 | Human | Henan, China | 2013 | D |  | MN510246 | Human | Hubei, China | 2015 | D |
| OM453383 | Human | Henan, China | 2017 | D |  | MN510069 | Human | Hubei, China | 2013 | D |  | OM451721 | Human | Hubei, China | 2014 | D |
| OM453296 | Human | Henan, China | 2017 | D |  | OM452922 | Human | Hubei, China | 2019 | D |  | MN510226 | Human | Henan, China | 2014 | D |
| OM453574 | Human | Henan, China | 2019 | D |  | MN510030 | Human | Henan, China | 2012 | D |  | OM452159 | Human | Henan, China | 2020 | D |
| OM453558 | Human | Henan, China | 2019 | D |  | OM452675 | Human | Henan, China | 2018 | D |  | OM452083 | Human | Henan, China | 2020 | D |
| OM453210 | Human | Henan, China | 2015 | D |  | OM452632 | Human | Henan, China | 2018 | D |  | OM451778 | Human | Henan, China | 2014 | D |
| OM453518 | Human | Henan, China | 2020 | D |  | OM452442 | Human | Henan, China | 2014 | D |  | OM451756 | Human | Henan, China | 2014 | D |
| OM453194 | Human | Henan, China | 2015 | D |  | MN510077 | Human | Henan, China | 2014 | D |  | OM452106 | Human | Henan, China | 2020 | D |
| OM453445 | Human | Henan, China | 2019 | D |  | OM452797 | Human | Henan, China | 2020 | D |  | OM452061 | Human | Henan, China | 2019 | D |
| OM453316 | Human | Henan, China | 2017 | D |  | MT114259 | Human | Hubei, China | 2014 | D |  | OM451874 | Human | Henan, China | 2016 | D |
| OM453232 | Human | Henan, China | 2016 | D |  | KC292320 | Human | Henan, China | 2012 | D |  | MN510245 | Human | Henan, China | 2015 | D |
| OM453567 | Human | Henan, China | 2019 | D |  | KY965101 | Human | Hubei, China | 2016 | D |  | OM451909 | Human | Hubei, China | 2017 | D |
| MN509922 | Human | Henan, China | 2014 | D |  | KY440773 | Human | Hubei, China | 2016 | D |  | KY965079 | Human | Hubei, China | 2016 | D |
| OM453380 | Human | Henan, China | 2018 | D |  | OM452776 | Human | Hubei, China | 2020 | D |  | OM451900 | Human | Henan, China | 2016 | D |
| OM453282 | Human | Henan, China | 2018 | D |  | OM452414 | Human | Henan, China | 2014 | D |  | OM452187 | Human | Henan, China | 2019 | D |
| OM452948 | Human | Henan, China | 2012 | D |  | OM452638 | Human | Hubei, China | 2018 | D |  | KU664015 | Human | Hubei, China | 2012 | D |
| OM453070 | Human | Henan, China | 2014 | D |  | OM452704 | Human | Henan, China | 2017 | D |  | MT114279 | Human | Hubei, China | 2012 | D |
| MN509895 | Human | Henan, China | 2013 | D |  | OM452759 | Human | Henan, China | 2019 | D |  | KF711918 | Human | Henan, China | 2012 | D |
| OM453490 | Human | Henan, China | 2020 | D |  | OM452747 | Human | Hubei, China | 2019 | D |  | OM452117 | Human | Henan, China | 2020 | D |
| OM453427 | Human | Henan, China | 2019 | D |  | OM452915 | Human | Hubei, China | 2019 | D |  | KF356519 | Human | Henan, China | 2011 | D |
| MN509957 | Human | Henan, China | 2016 | D |  | OM452920 | Human | Hubei, China | 2019 | D |  | KF356521 | Human | Henan, China | 2011 | D |
| ON402247 | Human | Henan, China | 2022 | D |  | KC292306 | Human | Henan, China | 2012 | D |  | OM452197 | Human | Hubei, China | 2019 | D |
| OM453042 | Human | Henan, China | 2013 | D |  | OM452696 | Human | Henan, China | 2017 | D |  | MN510282 | Human | Henan, China | 2017 | D |
| MT320805 | Human | Hubei, China | 2017 | D |  | MN510028 | Human | Henan, China | 2012 | D |  | OM452182 | Human | Henan, China | 2019 | D |
| KF356547 | Human | Henan, China | 2011 | D |  | OM452622 | Human | Henan, China | 2018 | D |  | OM452019 | Human | Hubei, China | 2018 | D |
| OM453456 | Human | Henan, China | 2020 | D |  | OM452823 | Human | Hubei, China | 2020 | D |  | OM452066 | Human | Hubei, China | 2019 | D |
| OM453615 | Human | Henan, China | 2019 | D |  | OM452524 | Human | Henan, China | 2015 | D |  | MN510210 | Human | Hubei, China | 2013 | D |
| OM453618 | Human | Henan, China | 2019 | D |  | MN510026 | Human | Henan, China | 2012 | D |  | OM451604 | Human | Henan, China | 2013 | D |
| OM453061 | Human | Henan, China | 2014 | D |  | MN510056 | Human | Henan, China | 2013 | D |  | MN995309 | Cat | Japan | 2019 | A |
| OM453136 | Human | Henan, China | 2014 | D |  | MN510175 | Human | Henan, China | 2018 | D |  | OM451930 | Human | Hubei, China | 2017 | D |
| OM453407 | Human | Henan, China | 2018 | D |  | OM452900 | Human | Hubei, China | 2019 | D |  | OM452121 | Human | Henan, China | 2020 | D |
| OM453377 | Human | Henan, China | 2017 | D |  | MN510151 | Human | Hubei, China | 2017 | D |  | MN510248 | Human | Henan, China | 2015 | D |
| OM453510 | Human | Henan, China | 2020 | D |  | OM452714 | Human | Hubei, China | 2018 | D |  | OM451918 | Human | Hubei, China | 2018 | D |
| OM453195 | Human | Henan, China | 2015 | D |  | OM452745 | Human | Hubei, China | 2019 | D |  | OM452225 | Human | Henan, China | 2019 | D |
| OM453288 | Human | Henan, China | 2017 | D |  | MN510080 | Human | Henan, China | 2015 | D |  | OM452009 | Human | Henan, China | 2018 | D |
| OM453107 | Human | Henan, China | 2014 | D |  | OM452278 | Human | Henan, China | 2013 | D |  | OM451669 | Human | Henan, China | 2013 | D |
| OM453315 | Human | Henan, China | 2018 | D |  | OM452410 | Human | Hubei, China | 2014 | D |  | OM451936 | Human | Henan, China | 2018 | D |
| MN509966 | Human | Henan, China | 2016 | D |  | MN510070 | Human | Hubei, China | 2013 | D |  | OM452119 | Human | Henan, China | 2020 | D |
| OM453429 | Human | Henan, China | 2019 | D |  | KF356532 | Human | Henan, China | 2011 | D |  | MN510207 | Human | Henan, China | 2013 | D |
| OM453253 | Human | Henan, China | 2016 | D |  | OM452765 | Human | Hubei, China | 2019 | D |  | MN510251 | Human | Henan, China | 2015 | D |
| MN509872 | Human | Henan, China | 2013 | D |  | OM452674 | Human | Henan, China | 2017 | D |  | OM451599 | Human | Henan, China | 2013 | D |
| OM453564 | Human | Henan, China | 2019 | D |  | OM452882 | Human | Hubei, China | 2019 | D |  | OM452183 | Human | Hubei, China | 2019 | D |
| MN509967 | Human | Henan, China | 2016 | D |  | OM452917 | Human | Henan, China | 2019 | D |  | OM452181 | Human | Hubei, China | 2019 | D |
| OM453128 | Human | Henan, China | 2014 | D |  | MN510149 | Human | Hubei, China | 2017 | D |  | OM452231 | Human | Hubei, China | 2019 | D |
| MN509892 | Human | Henan, China | 2013 | D |  | OM452285 | Human | Henan, China | 2013 | D |  | OM452176 | Human | Hubei, China | 2019 | D |
| OM453483 | Human | Henan, China | 2020 | D |  | OM452909 | Human | Henan, China | 2019 | D |  | OM452228 | Human | Hubei, China | 2019 | D |
| MN509951 | Human | Henan, China | 2015 | D |  | OM452875 | Human | Hubei, China | 2019 | D |  | OM451963 | Human | Henan, China | 2018 | D |
| OM453382 | Human | Henan, China | 2018 | D |  | OM452861 | Human | Hubei, China | 2020 | D |  | OM452223 | Human | Hubei, China | 2019 | D |
| OM453086 | Human | Henan, China | 2014 | D |  | MN510092 | Human | Henan, China | 2015 | D |  | OM452216 | Human | Hubei, China | 2019 | D |
| OM453200 | Human | Henan, China | 2015 | D |  | OM452818 | Human | Henan, China | 2020 | D |  | OM452221 | Human | Hubei, China | 2019 | D |
| OM453506 | Human | Henan, China | 2020 | D |  | OM452357 | Human | Henan, China | 2013 | D |  | OM452198 | Human | Henan, China | 2019 | D |
| OM453203 | Human | Henan, China | 2015 | D |  | OM452703 | Human | Henan, China | 2018 | D |  | OM451626 | Human | Henan, China | 2013 | D |
| OM453143 | Human | Henan, China | 2014 | D |  | MN510128 | Human | Hubei, China | 2016 | D |  | OM452003 | Human | Henan, China | 2017 | D |
| KC292338 | Human | Henan, China | 2011 | D |  | OM452820 | Human | Henan, China | 2020 | D |  | MN995310 | Cat | Japan | 2019 | A |
| OM453012 | Human | Henan, China | 2013 | D |  | OM452859 | Human | Henan, China | 2020 | D |  | OM451777 | Human | Henan, China | 2014 | D |
| KF711879 | Human | Henan, China | 2012 | D |  | OM452880 | Human | Hubei, China | 2019 | D |  | OM452124 | Human | Hubei, China | 2020 | D |
| OM453139 | Human | Henan, China | 2014 | D |  | OM452702 | Human | Henan, China | 2018 | D |  | OM452131 | Human | Hubei, China | 2020 | D |
| KU524075 | Human | Hubei, China | 2014 | D |  | OM452660 | Human | Henan, China | 2018 | D |  | OM451602 | Human | Henan, China | 2013 | D |
| HQ141595 | Human | Henan, China | 2010 | D |  | OM452924 | Human | Henan, China | 2019 | D |  | OM451751 | Human | Henan, China | 2014 | D |
| MN509932 | Human | Henan, China | 2015 | D |  | KF356530 | Human | Henan, China | 2011 | D |  | OM452184 | Human | Henan, China | 2019 | D |
| KC292340 | Human | Henan, China | 2011 | D |  | OM452654 | Human | Henan, China | 2018 | D |  | KT736094 | Human | Henan, China | 2015 | D |
| OM453604 | Human | Henan, China | 2019 | D |  | OM452848 | Human | Hubei, China | 2020 | D |  | MZ342903 | Cat | South Korea | 2020 | A |
| KC292337 | Human | Henan, China | 2011 | D |  | OM452896 | Human | Hubei, China | 2019 | D |  | KY440772 | Human | Hubei, China | 2015 | D |
| OM453076 | Human | Henan, China | 2014 | D |  | OM452876 | Human | Henan, China | 2019 | D |  | KP339918 | Human | Henan, China | 2014 | D |
| MN509871 | Human | Henan, China | 2013 | D |  | MN510126 | Human | Henan, China | 2016 | D |  | OM452060 | Human | Henan, China | 2019 | D |
| OM453331 | Human | Henan, China | 2018 | D |  | MN510035 | Human | Henan, China | 2012 | D |  | MN510243 | Human | Henan, China | 2015 | D |
| MN509941 | Human | Henan, China | 2015 | D |  | MN510160 | Human | Henan, China | 2018 | D |  | OM451945 | Human | Hubei, China | 2018 | D |
| OM453026 | Human | Henan, China | 2013 | D |  | MN510072 | Human | Hubei, China | 2013 | D |  | KU738906 | Human | Hubei, China | 2015 | D |
| OM453301 | Human | Henan, China | 2018 | D |  | OM452927 | Human | Hubei, China | 2019 | D |  | MT114289 | Human | Hubei, China | 2014 | D |
| MN509949 | Human | Henan, China | 2015 | D |  | MN510068 | Human | Henan, China | 2013 | D |  | MN510229 | Human | Henan, China | 2014 | D |
| MT114241 | Human | Hubei, China | 2012 | D |  | OM452397 | Human | Henan, China | 2014 | D |  | OM451687 | Human | Henan, China | 2014 | D |
| OM452982 | Human | Henan, China | 2013 | D |  | OM452760 | Human | Henan, China | 2019 | D |  | OM451741 | Human | Hubei, China | 2014 | D |
| KF711876 | Human | Henan, China | 2012 | D |  | MN510054 | Human | Henan, China | 2013 | D |  | OM451863 | Human | Henan, China | 2015 | D |
| MN509856 | Human | Henan, China | 2012 | D |  | OM452412 | Human | Henan, China | 2014 | D |  | OM452147 | Human | Henan, China | 2020 | D |
| OM453099 | Human | Henan, China | 2014 | D |  | MN510086 | Human | Henan, China | 2015 | D |  | HQ642768 | Human | Henan, China | 2010 | D |
| OM453113 | Human | Henan, China | 2014 | D |  | OM452574 | Human | Henan, China | 2016 | D |  | JF682778 | Human | Henan, China | 2010 | D |
| OM453284 | Human | Henan, China | 2017 | D |  | OM452886 | Human | Henan, China | 2019 | D |  | MN510313 | Human | Henan, China | 2018 | D |
| OM453378 | Human | Henan, China | 2018 | D |  | OM452805 | Human | Henan, China | 2020 | D |  | OM452096 | Human | Henan, China | 2020 | D |
| MN509863 | Human | Anhui, China | 2012 | D |  | KF711947 | Human | Henan, China | 2011 | D |  | MN510320 | Human | Henan, China | 2018 | D |
| OM453078 | Human | Henan, China | 2014 | D |  | OM452603 | Human | Hubei, China | 2017 | D |  | OM451564 | Human | Henan, China | 2012 | D |
| KF356541 | Human | Henan, China | 2011 | D |  | OM452911 | Human | Henan, China | 2019 | D |  | OM451801 | Human | Henan, China | 2015 | D |
| OM453147 | Human | Henan, China | 2014 | D |  | OM452867 | Human | Henan, China | 2019 | D |  | KP339885 | Human | Henan, China | 2014 | D |
| MN510017 | Human | Henan, China | 2018 | D |  | OM452816 | Human | Henan, China | 2020 | D |  | OM452036 | Human | Henan, China | 2017 | D |
| OM453612 | Human | Henan, China | 2019 | D |  | KF356531 | Human | Henan, China | 2011 | D |  | OM451978 | Human | Henan, China | 2018 | D |
| OM453320 | Human | Henan, China | 2018 | D |  | KF356533 | Human | Henan, China | 2011 | D |  | OM451939 | Human | Henan, China | 2018 | D |
| OM453299 | Human | Henan, China | 2017 | D |  | MN510122 | Human | Henan, China | 2016 | D |  | OM451798 | Human | Henan, China | 2015 | D |
| OM453587 | Human | Henan, China | 2019 | D |  | KY965096 | Human | Hubei, China | 2016 | D |  | OM451895 | Human | Hubei, China | 2016 | D |
| HQ171187 | Human | Hubei, China | 2010 | D |  | MT114249 | Human | Hubei, China | 2012 | D |  | OM452100 | Human | Henan, China | 2020 | D |
| OM453370 | Human | Henan, China | 2017 | D |  | KC292307 | Human | Henan, China | 2010 | D |  | OM452233 | Human | Henan, China | 2019 | D |
| OM453549 | Human | Henan, China | 2019 | D |  | KF356538 | Human | Henan, China | 2011 | D |  | OM452199 | Human | Henan, China | 2019 | D |
|  |  |  |  |  |  | OM452592 | Human | Henan, China | 2016 | D |  | OM452191 | Human | Henan, China | 2019 | D |
|  |  |  |  |  |  | MN510139 | Human | Henan, China | 2017 | D |  | OK423754 | Dog | South Korea | 2020 | A |
|  |  |  |  |  |  | OM452563 | Human | Henan, China | 2016 | D |  | OM452072 | Human | Henan, China | 2020 | D |
|  |  |  |  |  |  | MT114225 | Human | Liaoning, China | 2012 | D |  | MN510271 | Human | Henan, China | 2016 | D |
|  |  |  |  |  |  | JQ670931 | Human | Anhui, China | 2011 | D |  | OM451960 | Human | Henan, China | 2018 | D |
|  |  |  |  |  |  | MN510172 | Human | Henan, China | 2018 | D |  | OM452028 | Human | Henan, China | 2017 | D |
|  |  |  |  |  |  | MN510079 | Human | Henan, China | 2015 | D |  | OM452194 | Human | Henan, China | 2019 | D |
|  |  |  |  |  |  | MT114216 | Human | Liaoning, China | 2010 | D |  | OM451583 | Human | Henan, China | 2012 | D |
|  |  |  |  |  |  | MT114226 | Human | Liaoning, China | 2013 | D |  | OM452111 | Human | Henan, China | 2020 | D |
|  |  |  |  |  |  | MT114231 | Human | Liaoning, China | 2013 | D |  | JF682775 | Human | Henan, China | 2010 | D |
|  |  |  |  |  |  | MT114230 | Human | Liaoning, China | 2013 | D |  | OM451793 | Human | Henan, China | 2015 | D |
|  |  |  |  |  |  | KF358692 | Human | South Korea | 2012 | D |  | OM452202 | Human | Henan, China | 2019 | D |
|  |  |  |  |  |  | KY965097 | Human | Hubei, China | 2016 | D |  | OM451795 | Human | Henan, China | 2015 | D |
|  |  |  |  |  |  | HQ419231 | Human | Hubei, China | 2010 | D |  | OM452206 | Human | Henan, China | 2019 | D |
|  |  |  |  |  |  | MT114247 | Human | Hubei, China | 2011 | D |  | OM452144 | Human | Henan, China | 2020 | D |
|  |  |  |  |  |  | JQ317170 | Human | Jiangsu, China | 2010 | D |  | OM451898 | Human | Henan, China | 2016 | D |
|  |  |  |  |  |  | KC473538 | Goat | Jiangsu, China | 2012 | D |  | OM451803 | Human | Henan, China | 2015 | D |
|  |  |  |  |  |  | KY362352 | Human | Jiangsu, China | 2013 | D |  | OM452108 | Human | Henan, China | 2020 | D |
|  |  |  |  |  |  | KR230773 | Human | Jiangsu, China | 2013 | D |  | OM451885 | Human | Henan, China | 2016 | D |
|  |  |  |  |  |  | KY362354 | Human | Jiangsu, China | 2015 | D |  | KT380653 | Human | Henan, China | 2015 | D |
|  |  |  |  |  |  | KR230786 | Tick | Jiangsu, China | 2014 | D |  | OM452134 | Human | Henan, China | 2020 | D |
|  |  |  |  |  |  | KC505127 | Human | Jiangsu, China | 2012 | D |  | KP339931 | Human | Henan, China | 2014 | D |
|  |  |  |  |  |  | KR230772 | Human | Jiangsu, China | 2012 | D |  | OM451908 | Human | Henan, China | 2017 | D |
|  |  |  |  |  |  | KR230789 | Hedgehog | Jiangsu, China | 2014 | D |  | OM451947 | Human | Henan, China | 2018 | D |
|  |  |  |  |  |  | KR230778 | Human | Jiangsu, China | 2014 | D |  | OM451571 | Human | Hubei, China | 2012 | D |
|  |  |  |  |  |  | KR230787 | Tick | Jiangsu, China | 2014 | D |  | OM452017 | Human | Henan, China | 2018 | D |
|  |  |  |  |  |  | KY965108 | Human | Hubei, China | 2016 | D |  | KF711915 | Human | Henan, China | 2011 | D |
|  |  |  |  |  |  | KR706566 | Human | Hubei, China | 2014 | D |  | KF917438 | Human | Henan, China | 2013 | D |
|  |  |  |  |  |  | KC505133 | Human | Jiangsu, China | 2012 | D |  | KR230803 | Human | Jiangsu, China | 2014 | D |
|  |  |  |  |  |  | JQ317173 | Human | Jiangsu, China | 2010 | D |  | OM452201 | Human | Hubei, China | 2019 | D |
|  |  |  |  |  |  | KY362359 | Human | Jiangsu, China | 2013 | D |  | OM452128 | Human | Henan, China | 2020 | D |
|  |  |  |  |  |  | KY362346 | Human | Jiangsu, China | 2011 | D |  | KC505134 | Human | Jiangsu, China | 2012 | D |
|  |  |  |  |  |  | KY362357 | Human | Jiangsu, China | 2015 | D |  | OK423755 | Cat | South Korea | 2020 | A |
|  |  |  |  |  |  | KY362349 | Human | Jiangsu, China | 2011 | D |  | KC473539 | Goat | Jiangsu, China | 2012 | D |
|  |  |  |  |  |  | KR230783 | Human | Jiangsu, China | 2014 | D |  | KR230809 | Hedgehog | Jiangsu, China | 2014 | D |
|  |  |  |  |  |  | OM452786 | Human | Henan, China | 2020 | D |  | KY362330 | Human | Jiangsu, China | 2015 | D |
|  |  |  |  |  |  | OM452821 | Human | Henan, China | 2020 | D |  | OM160961 | Tick | South Korea | 2019 | A |
|  |  |  |  |  |  | OM452713 | Human | Henan, China | 2017 | D |  | OM160963 | Tick | South Korea | 2020 | A |
|  |  |  |  |  |  | OM452852 | Human | Henan, China | 2020 | D |  | KY965091 | Human | Hubei, China | 2016 | D |
|  |  |  |  |  |  | MN510059 | Human | Henan, China | 2013 | D |  | KR706565 | Human | Hubei, China | 2014 | D |
|  |  |  |  |  |  | OM452725 | Human | Henan, China | 2018 | D |  | JQ693011 | Human | Shandong, China | 2011 | D |
|  |  |  |  |  |  | OM452352 | Human | Henan, China | 2013 | D |  | OM160979 | Tick | South Korea | 2019 | A |
|  |  |  |  |  |  | OM452549 | Human | Henan, China | 2015 | D |  | MT559337 | Human | Anhui, China | 2020 | D |
|  |  |  |  |  |  | OM452635 | Human | Henan, China | 2017 | D |  | JQ693009 | Human | Shandong, China | 2011 | D |
|  |  |  |  |  |  | OM452513 | Human | Henan, China | 2015 | D |  | JQ693012 | Human | Shandong, China | 2011 | D |
|  |  |  |  |  |  | KC292319 | Human | Henan, China | 2012 | D |  | JQ693005 | Human | Shandong, China | 2011 | D |
|  |  |  |  |  |  | OM452280 | Human | Henan, China | 2013 | D |  | JQ693006 | Human | Shandong, China | 2011 | D |
|  |  |  |  |  |  | OM452767 | Human | Henan, China | 2020 | D |  | JQ693008 | Human | Shandong, China | 2011 | D |
|  |  |  |  |  |  | OM452344 | Human | Henan, China | 2013 | D |  | JQ693007 | Human | Shandong, China | 2011 | D |
|  |  |  |  |  |  | OM452921 | Human | Henan, China | 2019 | D |  | KY362324 | Human | Jiangsu, China | 2011 | D |
|  |  |  |  |  |  | OM452393 | Human | Henan, China | 2014 | D |  | KY362333 | Human | Jiangsu, China | 2015 | D |
|  |  |  |  |  |  | OM452533 | Human | Henan, China | 2015 | D |  | KY362335 | Human | Jiangsu, China | 2013 | D |
|  |  |  |  |  |  | OM452754 | Human | Henan, China | 2019 | D |  | KR230807 | Tick | Jiangsu, China | 2014 | D |
|  |  |  |  |  |  | OM452855 | Human | Henan, China | 2020 | D |  | KR230798 | Human | Jiangsu, China | 2014 | D |
|  |  |  |  |  |  | OM452260 | Human | Henan, China | 2012 | D |  | KY362331 | Human | Jiangsu, China | 2015 | D |
|  |  |  |  |  |  | OM452538 | Human | Henan, China | 2015 | D |  | KR230795 | Human | Jiangsu, China | 2013 | D |
|  |  |  |  |  |  | OM452634 | Human | Henan, China | 2018 | D |  | KR230793 | Human | Jiangsu, China | 2013 | D |
|  |  |  |  |  |  | OM452529 | Human | Henan, China | 2015 | D |  | KY362328 | Human | Jiangsu, China | 2013 | D |
|  |  |  |  |  |  | OM452743 | Human | Henan, China | 2017 | D |  | KR230792 | Human | Jiangsu, China | 2012 | D |
|  |  |  |  |  |  | OM452304 | Human | Hubei, China | 2013 | D |  | KR230802 | Human | Jiangsu, China | 2014 | D |
|  |  |  |  |  |  | KF711946 | Human | Henan, China | 2012 | D |  | KR230796 | Human | Jiangsu, China | 2013 | D |
|  |  |  |  |  |  | MN510063 | Human | Henan, China | 2013 | D |  | KC505128 | Human | Jiangsu, China | 2012 | D |
|  |  |  |  |  |  | OM452616 | Human | Henan, China | 2017 | D |  | OM452205 | Human | Henan, China | 2019 | D |
|  |  |  |  |  |  | OM452531 | Human | Henan, China | 2015 | D |  | JQ670933 | Human | Anhui, China | 2011 | D |
|  |  |  |  |  |  | OM452831 | Human | Henan, China | 2020 | D |  | OM452178 | Human | Henan, China | 2019 | D |
|  |  |  |  |  |  | OM452456 | Human | Henan, China | 2014 | D |  | MT114269 | Human | Liaoning, China | 2012 | D |
|  |  |  |  |  |  | OM452451 | Human | Henan, China | 2014 | D |  | OM452006 | Human | Henan, China | 2018 | D |
|  |  |  |  |  |  | OM452461 | Human | Henan, China | 2014 | D |  | OM451644 | Human | Henan, China | 2013 | D |
|  |  |  |  |  |  | MN510033 | Human | Henan, China | 2012 | D |  | OM451592 | Human | Henan, China | 2013 | D |
|  |  |  |  |  |  | OM452374 | Human | Henan, China | 2014 | D |  | MN510291 | Human | Henan, China | 2017 | D |
|  |  |  |  |  |  | OM452388 | Human | Henan, China | 2014 | D |  | OM451698 | Human | Henan, China | 2014 | D |
|  |  |  |  |  |  | HQ419229 | Human | Hubei, China | 2010 | D |  | OM451775 | Human | Henan, China | 2014 | D |
|  |  |  |  |  |  | ON402253 | Human | Henan, China | 2022 | D |  | KP339889 | Human | Henan, China | 2014 | D |
|  |  |  |  |  |  | KY965127 | Human | Hubei, China | 2016 | D |  | OM451636 | Human | Henan, China | 2013 | D |
|  |  |  |  |  |  | MT320803 | Human | Hubei, China | 2017 | D |  | OM451764 | Human | Henan, China | 2014 | D |
|  |  |  |  |  |  | OM452558 | Human | Henan, China | 2016 | D |  | OM451814 | Human | Henan, China | 2015 | D |
|  |  |  |  |  |  | MN510116 | Human | Henan, China | 2016 | D |  | OM452073 | Human | Henan, China | 2020 | D |
|  |  |  |  |  |  | OM452577 | Human | Henan, China | 2016 | D |  | OM451565 | Human | Henan, China | 2012 | D |
|  |  |  |  |  |  | OM452906 | Human | Henan, China | 2019 | D |  | OM451836 | Human | Henan, China | 2015 | D |
|  |  |  |  |  |  | OM452879 | Human | Henan, China | 2019 | D |  | OM451705 | Human | Henan, China | 2014 | D |
|  |  |  |  |  |  | OM452869 | Human | Henan, China | 2019 | D |  | OM451605 | Human | Henan, China | 2013 | D |
|  |  |  |  |  |  | OM452868 | Human | Henan, China | 2019 | D |  | OM451808 | Human | Henan, China | 2015 | D |
|  |  |  |  |  |  | OM452439 | Human | Henan, China | 2014 | D |  | OM451950 | Human | Henan, China | 2018 | D |
|  |  |  |  |  |  | OM452553 | Human | Henan, China | 2015 | D |  | OM451816 | Human | Henan, China | 2015 | D |
|  |  |  |  |  |  | OM452936 | Human | Henan, China | 2019 | D |  | OM452020 | Human | Henan, China | 2018 | D |
|  |  |  |  |  |  | OM452346 | Human | Henan, China | 2013 | D |  | MN510189 | Human | Henan, China | 2012 | D |
|  |  |  |  |  |  | OM452573 | Human | Henan, China | 2016 | D |  | MN510183 | Human | Henan, China | 2012 | D |
|  |  |  |  |  |  | OM452244 | Human | Henan, China | 2011 | D |  | KT736090 | Human | Henan, China | 2015 | D |
|  |  |  |  |  |  | OM452737 | Human | Henan, China | 2018 | D |  | MN510186 | Human | Henan, China | 2012 | D |
|  |  |  |  |  |  | OM452302 | Human | Henan, China | 2013 | D |  | MN510324 | Human | Henan, China | 2018 | D |
|  |  |  |  |  |  | OM452783 | Human | Henan, China | 2020 | D |  | OM452057 | Human | Henan, China | 2019 | D |
|  |  |  |  |  |  | MN510100 | Human | Henan, China | 2015 | D |  | OM451755 | Human | Henan, China | 2014 | D |
|  |  |  |  |  |  | OM452358 | Human | Henan, China | 2013 | D |  | MN510270 | Human | Henan, China | 2016 | D |
|  |  |  |  |  |  | OM452546 | Human | Henan, China | 2015 | D |  | OM451631 | Human | Henan, China | 2013 | D |
|  |  |  |  |  |  | OM452385 | Human | Henan, China | 2014 | D |  | OM451946 | Human | Henan, China | 2018 | D |
|  |  |  |  |  |  | OM452701 | Human | Henan, China | 2017 | D |  | OM452209 | Human | Henan, China | 2019 | D |
|  |  |  |  |  |  | OM452856 | Human | Henan, China | 2020 | D |  | HQ171192 | Human | Hubei, China | 2010 | D |
|  |  |  |  |  |  | MN510123 | Human | Henan, China | 2016 | D |  | OM451931 | Human | Henan, China | 2017 | D |
|  |  |  |  |  |  | OM452365 | Human | Henan, China | 2013 | D |  | OM452211 | Human | Henan, China | 2019 | D |
|  |  |  |  |  |  | OM452407 | Human | Henan, China | 2014 | D |  | OM452002 | Human | Henan, China | 2018 | D |
|  |  |  |  |  |  | OM452761 | Human | Henan, China | 2019 | D |  | HQ419242 | Human | Henan, China | 2010 | D |
|  |  |  |  |  |  | MN510022 | Human | Henan, China | 2011 | D |  | KP339895 | Human | Henan, China | 2014 | D |
|  |  |  |  |  |  | OM452621 | Human | Henan, China | 2018 | D |  | KP339905 | Human | Henan, China | 2014 | D |
|  |  |  |  |  |  | OM452398 | Human | Henan, China | 2014 | D |  | OM452234 | Human | Henan, China | 2019 | D |
|  |  |  |  |  |  | OM452290 | Human | Henan, China | 2013 | D |  | OM160981 | Tick | South Korea | 2020 | A |
|  |  |  |  |  |  | MT114255 | Human | Hubei, China | 2012 | D |  | OM451855 | Human | Henan, China | 2015 | D |
|  |  |  |  |  |  | KC292313 | Human | Henan, China | 2012 | D |  | OM451674 | Human | Henan, China | 2013 | D |
|  |  |  |  |  |  | OM452293 | Human | Henan, China | 2013 | D |  | KF711902 | Human | Henan, China | 2012 | D |
|  |  |  |  |  |  | OM452774 | Human | Henan, China | 2020 | D |  | KF711903 | Human | Henan, China | 2012 | D |
|  |  |  |  |  |  | MN510094 | Human | Henan, China | 2015 | D |  | KP339928 | Human | Henan, China | 2014 | D |
|  |  |  |  |  |  | MN510076 | Human | Henan, China | 2014 | D |  | KP339896 | Human | Henan, China | 2014 | D |
|  |  |  |  |  |  | MN510093 | Human | Henan, China | 2015 | D |  | KP339921 | Human | Henan, China | 2014 | D |
|  |  |  |  |  |  | OM452348 | Human | Henan, China | 2013 | D |  | KU361341 | Human | Hubei, China | 2013 | D |
|  |  |  |  |  |  | OM452671 | Human | Henan, China | 2018 | D |  | OM451962 | Human | Henan, China | 2018 | D |
|  |  |  |  |  |  | OM452520 | Human | Henan, China | 2015 | D |  | OM452204 | Human | Henan, China | 2019 | D |
|  |  |  |  |  |  | OM452339 | Human | Henan, China | 2013 | D |  | OM452180 | Human | Henan, China | 2019 | D |
|  |  |  |  |  |  | OM452646 | Human | Henan, China | 2018 | D |  | OM451684 | Human | Henan, China | 2014 | D |
|  |  |  |  |  |  | OM452750 | Human | Henan, China | 2019 | D |  | OM451589 | Human | Henan, China | 2013 | D |
|  |  |  |  |  |  | OM452903 | Human | Henan, China | 2019 | D |  | MN510301 | Human | Henan, China | 2017 | D |
|  |  |  |  |  |  | OM452641 | Human | Henan, China | 2018 | D |  | OM452240 | Human | Henan, China | 2019 | D |
|  |  |  |  |  |  | OM452885 | Human | Henan, China | 2019 | D |  | OM452170 | Human | Henan, China | 2019 | D |
|  |  |  |  |  |  | MN510090 | Human | Henan, China | 2015 | D |  | OM452237 | Human | Henan, China | 2019 | D |
|  |  |  |  |  |  | OM452458 | Human | Henan, China | 2014 | D |  | OM452015 | Human | Hubei, China | 2018 | D |
|  |  |  |  |  |  | MN510159 | Human | Henan, China | 2018 | D |  | OM452068 | Human | Henan, China | 2020 | D |
|  |  |  |  |  |  | OM452322 | Human | Henan, China | 2013 | D |  | KP339922 | Human | Henan, China | 2014 | D |
|  |  |  |  |  |  | KX302604 | Human | Anhui, China | 2015 | D |  | OM451621 | Human | Henan, China | 2013 | D |
|  |  |  |  |  |  | OM452316 | Human | Henan, China | 2013 | D |  | OM451670 | Human | Henan, China | 2013 | D |
|  |  |  |  |  |  | OM452772 | Human | Henan, China | 2020 | D |  | OM452001 | Human | Henan, China | 2017 | D |
|  |  |  |  |  |  | MN510048 | Human | Henan, China | 2012 | D |  | OM451830 | Human | Henan, China | 2015 | D |
|  |  |  |  |  |  | MN510037 | Human | Henan, China | 2012 | D |  | KP339894 | Human | Henan, China | 2014 | D |
|  |  |  |  |  |  | MN510046 | Human | Henan, China | 2012 | D |  | OM451722 | Human | Henan, China | 2014 | D |
|  |  |  |  |  |  | OM452715 | Human | Henan, China | 2018 | D |  | OM451651 | Human | Henan, China | 2013 | D |
|  |  |  |  |  |  | OM452310 | Human | Henan, China | 2013 | D |  | MN510286 | Human | Henan, China | 2017 | D |
|  |  |  |  |  |  | HQ141596 | Human | Henan, China | 2010 | D |  | OM451923 | Human | Henan, China | 2017 | D |
|  |  |  |  |  |  | OM452587 | Human | Henan, China | 2016 | D |  | OM451910 | Human | Henan, China | 2018 | D |
|  |  |  |  |  |  | KR080475 | Human | Hubei, China | 2014 | D |  | KP339898 | Human | Henan, China | 2014 | D |
|  |  |  |  |  |  | MT320809 | Human | Hubei, China | 2017 | D |  | OM451870 | Human | Henan, China | 2016 | D |
|  |  |  |  |  |  | OM452904 | Human | Henan, China | 2019 | D |  | OM451842 | Human | Henan, China | 2015 | D |
|  |  |  |  |  |  | OM452899 | Human | Henan, China | 2019 | D |  | OM452189 | Human | Henan, China | 2019 | D |
|  |  |  |  |  |  | OM452405 | Human | Henan, China | 2014 | D |  | KT721297 | Human | Henan, China | 2015 | D |
|  |  |  |  |  |  | KC292314 | Human | Henan, China | 2012 | D |  | OM451883 | Human | Henan, China | 2016 | D |
|  |  |  |  |  |  | KC292324 | Human | Henan, China | 2012 | D |  | OM452203 | Human | Henan, China | 2019 | D |
|  |  |  |  |  |  | OM452382 | Human | Henan, China | 2014 | D |  | MN510323 | Human | Henan, China | 2018 | D |
|  |  |  |  |  |  | OM452925 | Human | Henan, China | 2019 | D |  | OM451772 | Human | Henan, China | 2014 | D |
|  |  |  |  |  |  | OM452273 | Human | Henan, China | 2013 | D |  | MN510256 | Human | Henan, China | 2016 | D |
|  |  |  |  |  |  | KX302600 | Human | Anhui, China | 2015 | D |  | OM451970 | Human | Henan, China | 2018 | D |
|  |  |  |  |  |  | KF356529 | Human | Henan, China | 2011 | D |  | OM452084 | Human | Henan, China | 2020 | D |
|  |  |  |  |  |  | OM452467 | Human | Henan, China | 2014 | D |  | KT380657 | Human | Henan, China | 2015 | D |
|  |  |  |  |  |  | OM452319 | Human | Henan, China | 2013 | D |  | OM451980 | Human | Henan, China | 2018 | D |
|  |  |  |  |  |  | KX302603 | Human | Anhui, China | 2015 | D |  | OM451703 | Human | Henan, China | 2014 | D |
|  |  |  |  |  |  | MN510025 | Human | Anhui, China | 2012 | D |  | KF917428 | Human | Henan, China | 2013 | D |
|  |  |  |  |  |  | OM452923 | Human | Henan, China | 2019 | D |  | MN510239 | Human | Henan, China | 2014 | D |
|  |  |  |  |  |  | MN510078 | Human | Henan, China | 2014 | D |  | OM451768 | Human | Henan, China | 2014 | D |
|  |  |  |  |  |  | OM452775 | Human | Henan, China | 2020 | D |  | OM452021 | Human | Henan, China | 2018 | D |
|  |  |  |  |  |  | MN510117 | Human | Henan, China | 2016 | D |  | OM451859 | Human | Henan, China | 2015 | D |
|  |  |  |  |  |  | OM452659 | Human | Henan, China | 2018 | D |  | OM451683 | Human | Henan, China | 2013 | D |
|  |  |  |  |  |  | OM452312 | Human | Henan, China | 2013 | D |  | HQ171191 | Human | Hubei, China | 2010 | D |
|  |  |  |  |  |  | OM452756 | Human | Henan, China | 2019 | D |  | MN510185 | Human | Henan, China | 2012 | D |
|  |  |  |  |  |  | OM452324 | Human | Henan, China | 2013 | D |  | MN510273 | Human | Henan, China | 2016 | D |
|  |  |  |  |  |  | OM452800 | Human | Henan, China | 2020 | D |  | OM451955 | Human | Henan, China | 2018 | D |
|  |  |  |  |  |  | OM452695 | Human | Henan, China | 2018 | D |  | MN510300 | Human | Henan, China | 2017 | D |
|  |  |  |  |  |  | MN510170 | Human | Henan, China | 2018 | D |  | OM451647 | Human | Henan, China | 2013 | D |
|  |  |  |  |  |  | OM452289 | Human | Henan, China | 2013 | D |  | KP339909 | Human | Henan, China | 2014 | D |
|  |  |  |  |  |  | OM452870 | Human | Henan, China | 2019 | D |  | OM451840 | Human | Henan, China | 2015 | D |
|  |  |  |  |  |  | OM452933 | Human | Henan, China | 2019 | D |  | OM451560 | Human | Henan, China | 2011 | G |
|  |  |  |  |  |  | OM452619 | Human | Henan, China | 2017 | D |  | OM451718 | Human | Henan, China | 2014 | D |
|  |  |  |  |  |  | OM452908 | Human | Henan, China | 2019 | D |  | MN510238 | Human | Hubei, China | 2014 | D |
|  |  |  |  |  |  | MN510097 | Human | Henan, China | 2015 | D |  | MT114285 | Human | Hubei, China | 2012 | D |
|  |  |  |  |  |  | OM452853 | Human | Henan, China | 2020 | D |  | ON402259 | Human | Henan, China | 2022 | D |
|  |  |  |  |  |  | OM452341 | Human | Henan, China | 2013 | D |  | MN510214 | Human | Henan, China | 2013 | D |
|  |  |  |  |  |  | OM452287 | Human | Henan, China | 2013 | D |  | OM452045 | Human | Henan, China | 2019 | D |
|  |  |  |  |  |  | OM452700 | Human | Henan, China | 2018 | D |  | OM451596 | Human | Henan, China | 2013 | D |
|  |  |  |  |  |  | OM452827 | Human | Henan, China | 2020 | D |  | OM451624 | Human | Hubei, China | 2013 | D |
|  |  |  |  |  |  | OM452523 | Human | Henan, China | 2015 | D |  | OM451653 | Human | Henan, China | 2013 | D |
|  |  |  |  |  |  | KX302597 | Human | Anhui, China | 2015 | D |  | KY965075 | Human | Hubei, China | 2016 | D |
|  |  |  |  |  |  | KX302601 | Human | Anhui, China | 2015 | D |  | OM451848 | Human | Henan, China | 2015 | D |
|  |  |  |  |  |  | MN510034 | Human | Henan, China | 2012 | D |  | MN510193 | Human | Henan, China | 2012 | D |
|  |  |  |  |  |  | OM452877 | Human | Henan, China | 2019 | D |  | OM451642 | Human | Henan, China | 2013 | D |
|  |  |  |  |  |  |  |  |  |  |  |  | OM451822 | Human | Henan, China | 2015 | D |
|  |  |  |  |  |  |  |  |  |  |  |  | OM451802 | Human | Henan, China | 2015 | D |
|  |  |  |  |  |  |  |  |  |  |  |  | MN510178 | Human | Henan, China | 2011 | D |
|  |  |  |  |  |  |  |  |  |  |  |  | OM451570 | Human | Henan, China | 2012 | D |
|  |  |  |  |  |  |  |  |  |  |  |  | OM451844 | Human | Henan, China | 2015 | D |
|  |  |  |  |  |  |  |  |  |  |  |  | OM452055 | Human | Henan, China | 2019 | D |
|  |  |  |  |  |  |  |  |  |  |  |  | OM452222 | Human | Henan, China | 2019 | D |
