# Supplementary material for: Genomic diversity and evolution analysis of severe fever with thrombocytopenia syndrome in East Asia from 2010 to 2022
Source: Front Microbiol. 2023 Aug 21;14:1233693. doi: 10.3389/fmicb.2023.1233693 (PMC10476882; doi:10.3389/fmicb.2023.1233693)
Supplement: Supplementary file 1 [file Data_Sheet_1.zip › Supplementary Table S2.DOCX]

**Supplementary Table S2 Recombination events of L segment detected using the RDP4**

| Recombinant segment | Major parent | Minor parent | Tools |
| --- | --- | --- | --- |
| MT114235(Hubei) | KY965114(Hubei) | JQ733567(Hubei) | RGBMCST |
| KC292329(Henan) | OM453610(Henan) | MN509999(Henan) | RGBMCST |
| KC292349(Henan) | OM453362(Henan) | MN509967(Henan) | RGBMCST |
| MT320802(Hubei) | OM452994(Henan) | KF356547(Henan) | RGBMST |
| KF711863(Henan) | OM453009(Henan) | OM453577(Henan) | RGBMST |
| KF711880(Henan) | OM453577(Henan) | OM453027(Henan) | RGBMCST |
| KF711889(Henan) | OM453313(Henan) | KF356548(Henan) | RBMST |
| OM452957(Henan) | OM453282(Henan) | MT005240(Shandong) | RBMCST |
| OM453009(Henan) | OM453347(Henan) | OM453427(Henan) | RGBMCST |
| OM453596(Henan) | OM453444(Hubei) | OM453477(Henan) | RGMCST |
| OM453266(Henan) | MN509918(Henan) | OM453594(Hubei) | RGBMCST |
| OM453577(Henan) | MN509843(Henan) | OM453258(Henan) | RGBMCST |
| KC292348(Henan) | KC292349(Henan) | MN509967(Henan) | GBMCST |
| MT413432(Hubei,Tick) | MT005236(Shandong) | ^#^ UNKNOWN | RGBMCST |
| OM453018(Henan) | MT005222(Shandong) | OM453320(Henan) | RGMCST |
| OM452994(Henan) | JQ733564(Hubei) | OM453288(Henan) | RGBMCST |
| OM453580(Henan) | MN509843(Henan) | OM453101(Henan) | RGBMCST |
| KC292336(Henan) | KC292350(Henan) | MN509929(Henan) | RGBMCST |
| KR698352(Zhejiang) | MZ773028(Zhejiang) | MZ773016(Zhejiang) | RGBMCST |
| AB983501(Japan) | AB817983(Japan) | AB983519(Japan) | RGBCST |
| OM453602(Henan) | MN510017(Henan) | KC292348(Hennan) | RGBMCT |
| MT005206(Shandong) | MT005238(Shandong) | KY362303(Jiangsu) | RGST |
| KR017835(Zhejiang) | KR017843(Zhejiang) | KR698348(Zhejiang) | RGBMCST |
| OM453579(Hubei) | MN509843(Henan) | OM453362(Henan) | RGBMCT |
| OM453562(Henan) | MN509843(Henan) | KC292348(Hennan) | RGBST |
| OM453270(Henan) | OM452960(Henan) | MN509932(Henan) | RGBMST |
| OM453616(Hubei) | KF356542(Henan) | OM453477(Henan) | RBMCT |
| KR698352(Zhejiang) | AB983500(Japan) | MZ773016(Zhejiang) | RGBMCST |
| KR698352(Zhejiang) | MZ773028(Zhejiang) | MZ773015(Zhejiang) | RGBT |
| MT005226(Shandong) | OM453367(Henan) | MT005213(Shandong) | GBMCST |
| MT522608(Anhui) | KY362307(Jiangsu) | JQ670934(Anhui) | RGMT |
| KC292336(Henan) | MN509967(Henan) | MN509867(Henan) | MST |
| OM453591(Henan) | MN509843(Henan) | OM453610(Henan) | RGB |
| MN509843(Henan) | KC292346(Henan) | ^#^ UNKNOWN | RGST |
| OM453017(Henan) | MN510013(Henan) | ^#^ UNKNOWN | RBS |
| KY362305(Jiangsu) | KY362294(Jinagsu) | KR706567(Hubei) | RGB |
| MT320808(Hubei) | MN509856(Henan) | OM453309(Henan) | RGBMCST |
| OM453027(Henan) | OM453610(Henan) | OM452979(Henan) | RGBMCST |
| KC292350(Henan) | OM453548(Henan) | OM453594(Hubei) | RMST |
| OM453594(Hubei) | OM453504(Hubei) | OM453080(Henan) | RGBMCST |
| OM453599(Hubei) | OM453601(Hubei) | OM453477(Henan) | RGBMCST |
| OM453547(Henan) | HQ171187(Hubei) | OM453129(Henan) | RGT |
| KF356542(Henan) | OM453337(Henan) | ^#^ UNKNOWN | GBT |
| KY965126(Hubei) | KC473537(Jiangsu,Goat) | MT005238(Shandong) | RGBT |
| OM453244(Henan) | MN509867(Henan) | MN509871(Henan) | BMCST |
| OM453583(Henan) | OM453567(Henan) | MT114245(Hubei) | MCS |
| OM453477(Henan) | OM453204(Henan) | KF356541(Henan) | RGBMCST |
| OM453548(Henan) | HQ171187(Hubei) | MT005223(Shandong) | RGMCT |
| MN509867(Henan) | KC292346(Henan) | ^#^ UNKNOWN | MCS |
| OM453432(Henan) | OM453204(Henan) | KF356541(Henan) | RGBMCST |
| OM452962(Henan) | OM453504(Hubei) | OM453549(Henan) | RGBT |
| OM453432(Henan) | OM453204(Henan) | KF356541(Henan) | RGBMCST |
| OM452960(Henan) | OM453204(Henan) | KF356541(Henan) | RGBMCST |
| KC292346(Henan) | OM453584(Henan) | OM453427(Henan) | RGB |

# Unknown indicates that the potential parental sequences were detected with low confidence. R means RDP; G means GENECONV; B means BootScan;

M means MaxChi; C means Chimaera; S means SiScan; T means 3Seq.
