# Supplementary material for: Genomic diversity and evolution analysis of severe fever with thrombocytopenia syndrome in East Asia from 2010 to 2022
Source: Front Microbiol. 2023 Aug 21;14:1233693. doi: 10.3389/fmicb.2023.1233693 (PMC10476882; doi:10.3389/fmicb.2023.1233693)
Supplement: Supplementary file 1 [file Data_Sheet_1.zip › Supplementary Table S3.DOCX]

**Supplementary Table S3 Recombination events of M segment detected using the RDP4**

| Recombinant segment | Major parent | Minor parent | Tools |
| --- | --- | --- | --- |
| KY362353(Jiangsu) | KY362345(Jiangsu) | KY362348(Jiangsu) | RGBMCST |
| MT522609(Anhui) | JQ670930(Anhui) | KY362349(Jiangsu) | RGBMST |
| OM452391(Henan) | MN510159(Henan) | OM452584(Henan) | RBMST |
| KY965103(Hubei) | OM452307(Henan) | KY965105(Hubei) | RBMST |
| KF711945(Henan) | KF711928(Henan) | HQ642767(Henan) | RBMST |
| KF356535(Henan) | KF711923(Henan) | HQ642767(Henan) | MCST |
| KR230776(Jiangsu) | KY362354(Jiangsu) | KR230771(Jiangsu) | RGBMCST |
| KR017854(Zhejiang) | KR017858(Zhejiang) | KR017855(Zhejiang) | GBMCST |
| KF711930(Henan) | OM452630(Henan) | OM452501(Henan) | RGBMCST |
| OM452787(Henan) | KR017860(Zhejiang) | MT114249(Hubei) | RGBMCST |
| MN510060(Henan) | HQ642767(Henan) | OM452259(Henan) | RGBMCST |
| KR017853(Zhejiang) | KR017855(Zhejiang) | KR230781(Jiangsu) | RBMCST |
| OM452259(Henan) | MN510027(Henan) | OM452768(Henan) | RGMCST |
| OM452332(Henan) | OM452580(Henan) | OM452307(Henan) | RGBMCST |
| KY965094（Hubei) | HQ141596(Henan) | MT005282(Shandong) | MST |
| KR698339(Zhejiang) | MZ773030(Zhejiang) | MZ773043(Zhejiang) | RGBMCST |
| KC292310(Henan) | OM452508(Henan) | OM452857(Henan) | RGBMCST |
| KR698339(Zhejiang) | LC570785(Japan, Dog) | MZ773043(Zhejiang) | RGST |
| KT890281(Jilin, Tick) | KC505145(Jiangsu) | KX302599(Anhui) | RGBMCS |
| OM452307(Henan) | MN510159(Henan) | OM452768(Henan) | GBT |
| OM452837(Henan) | UNKNOWN^#^ | OM452341(Henan) | RGBST |
| KY965128(Hubei) | MN510037(Henan) | UNKNOWN^#^ | RBT |
| HQ642767(Henan) | MN510037(Henan) | UNKNOWN^#^ | RGBT |
| OM452613(Hubei) | MN510087(Henan) | OM452573(Henan) | RGBT |
| KY965100(Hubei) | MT320800(Hubei) | KY965093(Hubei) | RGT |
| MN510084(Henan) | OM452630(Henan) | KC292310(Henan) | RGBMST |
| OM452726(Henan) | MN510071(Hubei) | OM452891(Henan) | RGBT |
| KF711931(Henan) | MN510159(Henan) | JQ733563(Hubei) | RGB |
| OM452508(Henan) | HQ141596(Henan) | OM452340(Henan) | GBT |
| MN510047(Hennan) | OM452508(Henan) | MN510087(Henan) | RGBT |
| KY362348(Jiangsu) | KY362349(Jiangsu) | KR230771(Jiangsu) | RGBST |
| KY965093(Hubei) | MT005248(Shandong) | KY965102(Hubei) | RGBT |
| OM452355(Henan) | OM452584(Henan) | KX302603(Anhui) | GBT |
| KR230782(Jiangsu) | KC473538(Jiangsu, Goat) | KR230780(Jiangsu) | GMT |
| KY362355(Jiangsu) | KC473538(Jiangsu, Goat) | KR230780(Jiangsu) | RMT |
| KR230775(Jiangsu) | KC473538(Jiangsu, Goat) | KR230771(Jiangsu) | RBMT |

# Unknown indicates that the potential parental sequences were detected with low confidence. R means RDP; G means GENECONV; B means BootScan;

M means MaxChi; C means Chimaera; S means SiScan; T means 3Seq.
