# Supplementary material for: Genomic diversity and evolution analysis of severe fever with thrombocytopenia syndrome in East Asia from 2010 to 2022
Source: Front Microbiol. 2023 Aug 21;14:1233693. doi: 10.3389/fmicb.2023.1233693 (PMC10476882; doi:10.3389/fmicb.2023.1233693)
Supplement: Supplementary file 1 [file Data_Sheet_1.zip › Supplementary Table S4.DOCX]

**Supplementary Table S4 Recombination events of S segment detected using the RDP4**

| Recombinant segment | Major parent | Minor parent | Tools |
| --- | --- | --- | --- |
| JQ693010(Shandong) | KF917448(Hubei) | KY362331(Jiangsu) | RGBMST |
| KU664010(Hubei) | KP339936(Henan) | MT114286(Hubei) | BMST |
| KR698328(Zhejiang) | KR698325(Zhejiang) | MZ561690(Zhejiang) | RGBMCST |
| KU664009(Hubei) | UNKNOWN^#^ | MT114288(Hubei) | RBMT |
| JQ693001(Shandong) | JQ693004(Shandong) | JQ693002(Shandong, Sheep) | MST |
| OM451759(Henan) | MN510215(Henan) | MN510288(Hubei) | RGBCST |
| JQ693002(Shandong, Sheep) | OM452212(Henan) | JQ693013(Shandong) | BMS |
| KY362321(Jiangsu) | KR230804(Jiangsu) | UNKNOWN^#^ | RGBT |
| OM451976(Henan) | KF356519(Henan) | KU738905(Hubei) | RGMCT |
| OM452238(Hubei) | MN510183(Henan) | KR230791(Jiangsu) | RGST |
| OM451720(Henan) | OM451726(Henan) | MN510288(Hubei) | RGBST |

# Unknown indicates that the potential parental sequences were detected with low confidence. R means RDP; G means GENECONV; B means BootScan;

M means MaxChi; C means Chimaera; S means SiScan; T means 3Seq.
