# Supplementary figures and images for: Genomic diversity and evolution analysis of severe fever with thrombocytopenia syndrome in East Asia from 2010 to 2022
Source: Front Microbiol. 2023 Aug 21;14:1233693. doi: 10.3389/fmicb.2023.1233693 (PMC10476882; doi:10.3389/fmicb.2023.1233693)

A


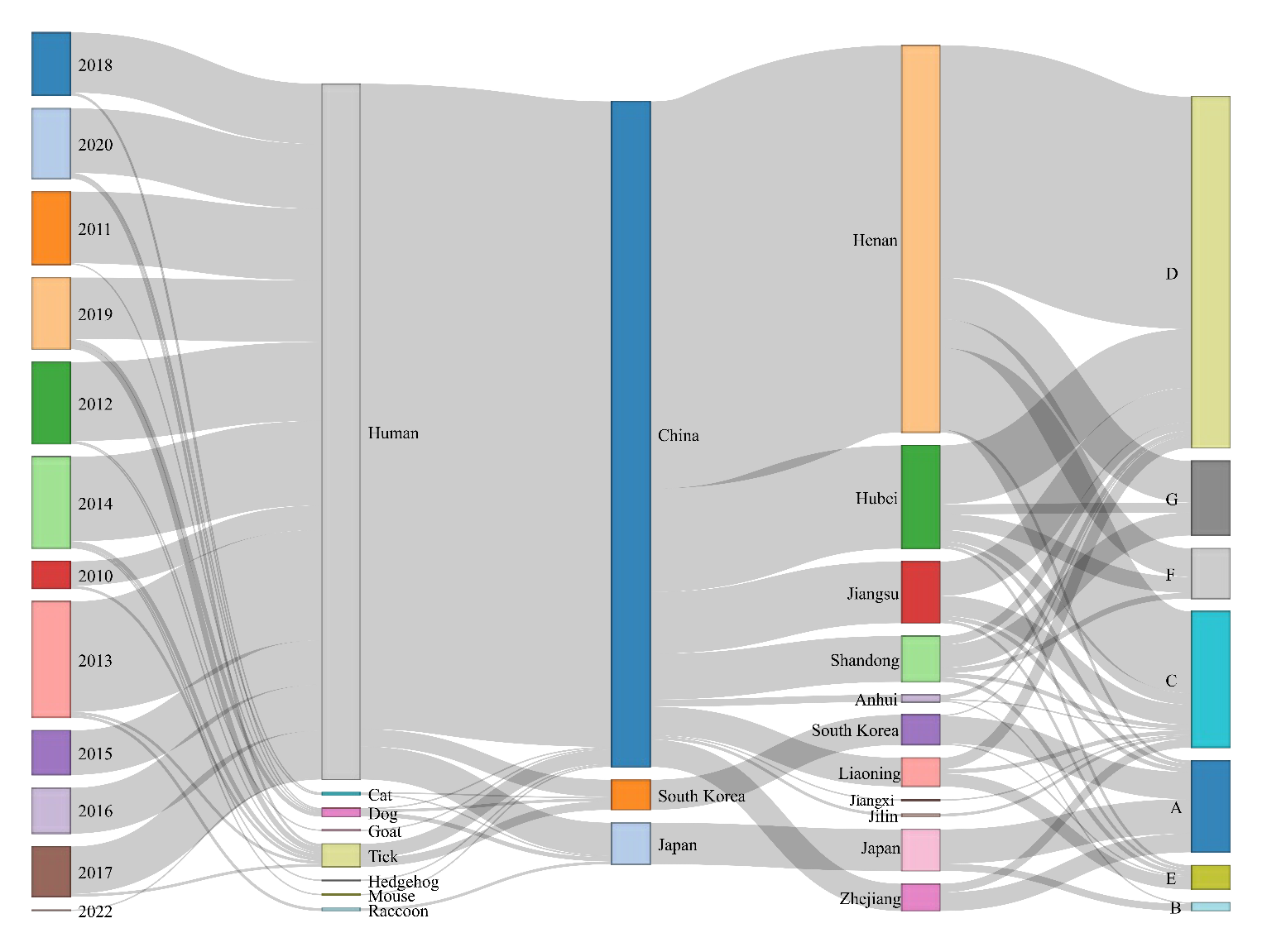


B


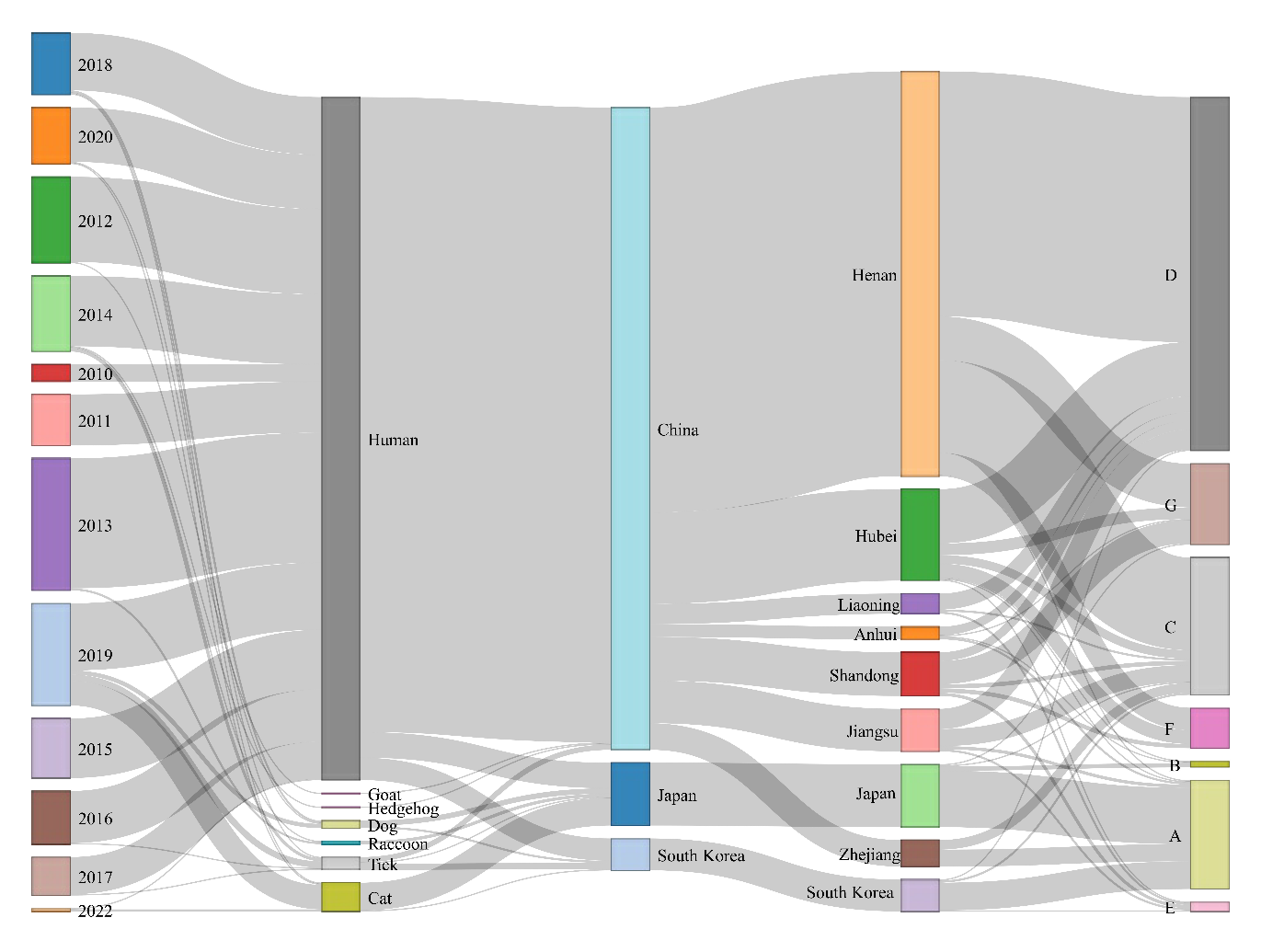


C


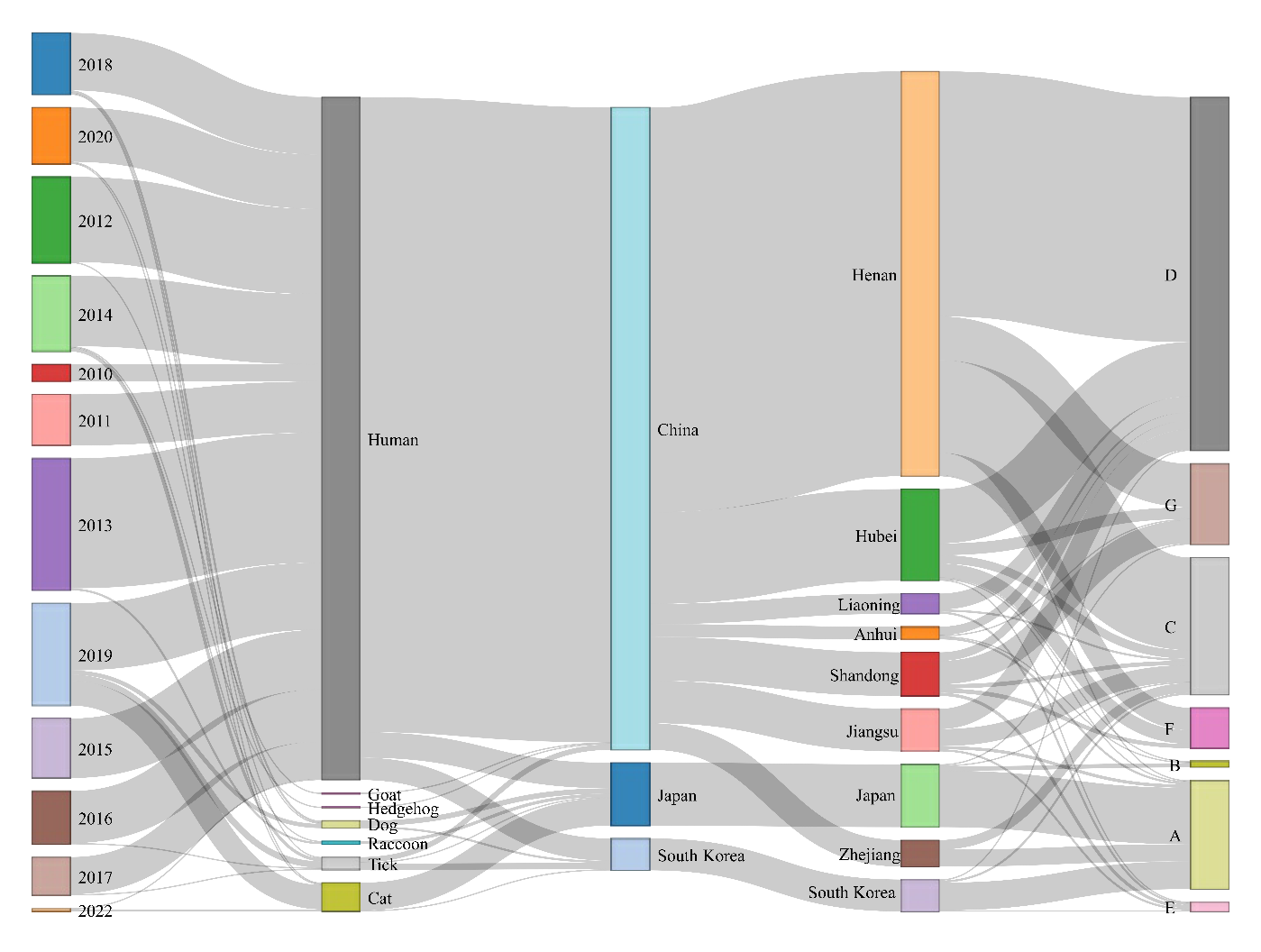


Supplementary Figure S1 The epidemiological information of L (A), M (B), and S (C) segments.

Supplement: Supplementary file 1 [file Data_Sheet_1.zip › Supplementary Figure S1.DOCX]
